# The Potential Mechanisms of High-Velocity, Low-Amplitude, Controlled Vertebral Thrusts on Neuroimmune Function: A Narrative Review

**DOI:** 10.3390/medicina57060536

**Published:** 2021-05-27

**Authors:** Heidi Haavik, Imran Khan Niazi, Nitika Kumari, Imran Amjad, Jenna Duehr, Kelly Holt

**Affiliations:** 1 Centre for Chiropractic Research, New Zealand College of Chiropractic, Auckland 1060, New Zealand; heidi.haavik@nzchiro.co.nz (H.H.); Nitika.kumari@nzchiro.co.nz (N.K.); imran.amjad@nzchiro.co.nz (I.A.); Jenna.Duehr@nzchiro.co.nz (J.D.); 2 Faculty of Health & Environmental Sciences, Health & Rehabilitation Research Institute, AUT University, Auckland 0627, New Zealand; 3 Department of Health Science and Technology, Aalborg University, 9220 Aalborg, Denmark; 4 Faculty of Rehabilitation and Allied Health Sciences, Riphah International University, Islamabad 46000, Pakistan

**Keywords:** high-velocity, low-amplitude thrust, HVLA, chiropractic, spinal manipulation, central nervous system, prefrontal cortex, immune system, endocrine system

## Abstract

The current COVID-19 pandemic has necessitated the need to find healthcare solutions that boost or support immunity. There is some evidence that high-velocity, low-amplitude (HVLA) controlled vertebral thrusts have the potential to modulate immune mediators. However, the mechanisms of the link between HVLA controlled vertebral thrusts and neuroimmune function and the associated potential clinical implications are less clear. This review aims to elucidate the underlying mechanisms that can explain the HVLA controlled vertebral thrust--neuroimmune link and discuss what this link implies for clinical practice and future research needs. A search for relevant articles published up until April 2021 was undertaken. Twenty-three published papers were found that explored the impact of HVLA controlled vertebral thrusts on neuroimmune markers, of which eighteen found a significant effect. These basic science studies show that HVLA controlled vertebral thrust influence the levels of immune mediators in the body, including neuropeptides, inflammatory markers, and endocrine markers. This narravtive review discusses the most likely mechanisms for how HVLA controlled vertebral thrusts could impact these immune markers. The mechanisms are most likely due to the known changes in proprioceptive processing that occur within the central nervous system (CNS), in particular within the prefrontal cortex, following HVLA spinal thrusts. The prefrontal cortex is involved in the regulation of the autonomic nervous system, the hypothalamic–pituitary–adrenal axis and the immune system. Bi-directional neuro-immune interactions are affected by emotional or pain-related stress. Stress-induced sympathetic nervous system activity also alters vertebral motor control. Therefore, there are biologically plausible direct and indirect mechanisms that link HVLA controlled vertebral thrusts to the immune system, suggesting HVLA controlled vertebral thrusts have the potential to modulate immune function. However, it is not yet known whether HVLA controlled vertebral thrusts have a clinically relevant impact on immunity. Further research is needed to explore the clinical impact of HVLA controlled vertebral thrusts on immune function.

## 1. Introduction

The COVID-19 pandemic has resulted in significant global morbidity and mortality [1,2], especially amongst individuals who are immunocompromised, such as older people and those with underlying medical conditions [2,3]. The current pandemic and other infectious diseases have led to a global search for effective vaccines and therapeutics to help boost immunity [4,5,6]. Health promotion models that aim to help improve host immunity are being developed [1,7]. These often incorporate approaches such as physical activity and exercise [1,7], nutrition [8,9], and a focus on mindfulness and mental health [10,11,12]. Over recent decades, healthcare providers have increasingly embraced a biopsychosocial model of healthcare that has incorporated approaches such as these to healthcare promotion [13,14]. One such healthcare modality that has been shown to influence neuroimmune chemical concentrations over the past two decades is manual or mechanically assisted application of specific high-velocity, low amplitude (HVLA), controlled vertebral thrusts (also known as chiropractic adjustments or spinal manipulation) [15,16,17,18,19,20,21,22,23,24,25,26,27,28,29,30,31,32]. Such HVLA thrusts delivered to spinal segments appear to improve vertebral column motor control by bombarding the central nervous system (CNS) with mechanoreceptive input from paraspinal tissues surrounding the segment, in particular, from the deep intervertebral paraspinal muscles [33,34,35,36,37]. There are known maladaptive plastic changes in the deep paraspinal muscles following a spinal injury [38,39,40,41,42,43]. Rapid atrophy due to neural inhibition [39,40], the development of muscle fibrosis, extensive fatty infiltration, and changes in muscle fibre types have all been found within the deep paraspinal muscles at various time-frames after a spinal injury [38,41,42,43,44]. The rapid and progressive degeneration of the cervical multifidus muscles has also been found to occur after cervical spine injuries such as whiplash, which include fatty infiltration of these deep paraspinal muscles of the neck [45,46]. These local paraspinal muscle changes coincide with ‘smudging’ within the primary sensorimotor cortices [47,48], and has led scientists to conclude that disrupted or reduced proprioceptive signalling from deep paraspinal muscles likely plays a pivotal role in driving long-term cortical reorganisation and changes in the top-down control of the sensorimotor systems and that this plays a vital role in driving the recurrence and chronicity of back pain [49]. Thus, the sensory (proprioceptive) information from deep paraspinal muscles around a dysfunctional vertebral motion segment, which for the purposes of this review will be referred to as a central segmental motor control (CSMC) problem (also known as a vertebral subluxation or joint dysfunction), is thought to be the driving factor in the widespread maladaptive neuroplastic changes within the CNS responsible for altered/poor vertebral column motor control and/or the development of chronic spinal pain syndromes. 

There is clear evidence that maladaptive dysfunction of the deep paraspinal muscles can occur [38,39,40,41,42,43,45,46]. This parspinal muscle dysfunction surrounding a CSMC problem likely reduces the ability of the CNS to accurately perceive what is going on at that level of the vertebral column. Over time it may be why there is a blurring of the sensorimotor cortical areas [47,48], and this is likely to lead to poor vertebral motor control, maintaining the CSMC problem in existence. As HVLA thrusts delivered to spinal segments have been shown to bombard the CNS with mechanoreceptive input from the deep intervertebral paraspinal muscles around the segment [33,34,35,36,37], this is the likely mechanism by which HVLA spinal thrusts improve spinal function and CNS motor control of the spine. However, what is less well understood is the mechanisms by which spinal HVLA thrusts can also impact the immune system, which recent evidence suggests occurs [15,16,17,18,19,20,21,22,23,24,25,26,27,28,29,30,31,32]. Therefore, the purpose of this narrative review is to explore the evidence that does exist for HVLA spinal thrusts impacting immune function and to discuss the most likely mechanisms for this impact. This review will also cover the limitations of this body of research, and highlight what (if any) implications this has on clinical care and will discuss future research needs.

## 2. Materials and Methods

The following databases were searched for studies published up until April 2021: CINAHL, MEDLINE, SPORTDiscus, Web of Science, Index to Chiropractic Literature, and Scopus. The search strategy (Supplementary S1) included a combination of key search terms, such as immune*, endocrine*, white blood cells, CD4, lymphocyte, chiropractic, spinal manipulation, osteopath*, manual therapy, high-velocity low-amplitude thrust, and spinal adjustment. In addition, relevant search terms from recent narrative and systematic reviews were also included to identify additional studies. The reference list of recent systematic reviews and meta-analyses were also searched. Full-text articles published in peer-reviewed English journals were included. Studies were excluded if they were conference papers, commentaries, letters, books, and theses. 

## 3. Results

Research investigating the effect of HVLA controlled vertebral thrusts on neuroimmune function and its potential mechanisms were extracted from the articles retrieved from the literature search (see Supplementary S2). 

### 3.1. Research Investigating a Link between HVLA Controlled Vertebral Thrusts and Immune Function

Twenty-three published papers were found that explored the impact of HVLA controlled vertebral thrusts on neuroimmune markers (Supplementary S2) [15,16,17,18,19,20,21,22,23,24,25,26,27,28,29,30,31,32,50,51,52,53,54], of which eighteen found a significant effect (non shaded rows in Supplementary S2) [15,16,17,18,19,20,21,22,23,24,25,26,27,28,29,30,31,32]. These basic science studies will be discussed below and show that HVLA controlled vertebral thrusts can influence the levels of immune mediators in the body, including neuropeptides, inflammatory markers, and endocrine markers [15,16,17,18,19,20,21,22,23,24,25,26,27,28,29,30,31,32]. The potential mechanisms for this impact will be discussed below in Section 3.2.

#### 3.1.1. Effect of HVLA Controlled Vertebral Thrusts on Immune Mediators

A number of basic science studies have been published that have evaluated the effect of HVLA controlled vertebral thrusts on various immune mediators, including neuropeptides (like neurotensin, oxytocin, and substance P), inflammatory markers (like tumour necrosis factor (TNF) and interleukins (IL)), and endocrine markers (like cortisol and epinephrine [15,16,17,18,19,20,21,22,23,24,25,26,27,28,29,30,31,32,50,51,52,53,54]. There is moderate level evidence that HVLA controlled vertebral thrusts impact these immune mediators [55,56]. A systematic review and meta-analysis, that included healthy and/or symptomatic subjects, showed that HVLA controlled vertebral thrusts can influence neuropeptides and inflammatory biomarkers that are important biochemicals associated with the function of the immune system [55].

There is moderate quality evidence, from one study, that suggests that HVLA controlled vertebral thrusts increase neurotensin and oxytocin levels [29,55]. An RCT involving 30 healthy individuals reported that, compared to a control, both cervical and thoracic HVLA controlled vertebral thrusts increased neurotensin and oxytocin levels immediately, but not two hours, after the intervention [29]. Although this finding suggests that HVLA controlled vertebral thrusts have an influence on immune function, it is not known if this is clinically relevant, as the changes were only short term. The study was of moderate quality and lacked blinding of participants and investigators, which is common in manual therapy studies. In contrast, an RCT of 28 women with non-specific mechanical neck pain found no significant difference in neurotensin, oxytocin, orexin A, and cortisol levels between the cervical HVLA controlled vertebral thrust and sham group. However, there was significant within group difference in neurotensin, oxytocin, and orexin A levels in the intervention group only [32]. Neurotensin, a neuropeptide that is abundant in the CNS and the GI tract, is an important modulator of neuroendocrine function [57,58,59]. It has been shown to have an anti-inflammatory role that involves downregulating the activation of the pro-inflammatory cytokines, such as IL-6, tumour necrosis factor alpha (TNF-α), and IL-10 [60]. It is also involved in the regulation of the HPA axis as it is responsible for increasing corticosterone and decreasing luteinising hormone, TSH and T4 production. Oxytocin plays an essential role in the hypothalamic neuroendocrine system, which is important in regulating immune function by regulating hormone secretion from the pituitary gland [61]. The oxytocin secreting cells can integrate neural, endocrine, metabolic, and immune information and play a pivotal role in the development and functions of the immune system [61]. In addition, it promotes the development of thymus and bone marrow, performs immune surveillance, strengthens immune defence, and maintains immune homeostasis [61]. It can also inhibit inflammation by suppressing pro-inflammatory cytokines, exert antibiotic-like effects, promote wound healing and regeneration, and suppress stress-associated immune disorders [61]. It is released from the posterior pituitary in response to sexual stimulation, uterine dilatation, nursing, but also in some situations, due to stress [62]. 

There is low-quality evidence suggesting that HVLA controlled vertebral thrusts increase levels of substance P [55]. As compared to a control, HVLA controlled vertebral thrusts increased substance P in healthy individuals immediately after the intervention [15,19,28], but not after a delay [19,28]. Substance P is a neuropeptide that can modulate immune cell proliferation rates and cytokine production [63] and alter the immune functions of activated microglia and astrocytes [64]. Substance P also plays a major role in pain perception and neurogenic inflammation [63]. However, the evidence regarding substance P is not all positive, because it has also been shown to be involved with secondary pathophysiology after traumatic brain injuries [65]. It has also been shown to be involved in bone marrow fibrosis and tumor cell proliferation and to exacerbate inflammation in people with a variety of conditions [63]. Studies on animals have even shown that substance P induces antisocial aggressiveness and regulates emotional behaviour such as depression and anxiety [66]. In human studies, it has been shown to be involved in negative emotions and in addictive behaviours [66].

A recent narrative review of randomized and non-randomized controlled trials concluded that the evidence for the effect of HVLA controlled vertebral thrusts on cortisol level in healthy or symptomatic individuals is inconsistent and limited by bias from various factors (for details refer to Colombi, 2019) [56]. Cortisol or glucocorticoids are important modulators of the immune response because they act to suppress cytokines and inflammation [67,68,69,70,71]. Suppression of inflammation by cortisol depends on whether the stress is acute or chronic [72]. Following the initial pro-inflammatory sympathetic response to acute stress, there is an increase in cortisol levels that reduces the inflammation, inhibits non-vital organs, and mobilizes glucose to regulate the acute stress [72,73]. However, chronic exposure to stress causing prolonged and excessive surges of cortisol depletes the levels of cortisol that results in increased inflammatory response [74,75,76,77]. 

Interleukins are a group of cytokines that play a major role in immune function [71]. They are cytokines that are released by immune cells when immune cells detect pathogens or tissue injury or in response to stress or trauma [71,78,79]. Some of them are known to be strongly pro-inflammatory, such as IL-1 beta and TNF-α. Others are known to be anti-inflammatory, for example IL-2, IL-4, and IL-10. However, many interleukins can be both pro- and anti-inflammatory, particularly IL-6. There is evidence that HVLA controlled vertebral thrusts decrease pro-inflammatory IL-1 beta and TNF-α [19], and increase anti-inflammatory cytokines like IL-2 [20,21]. There is moderate quality evidence that spinal manipulation influences interleukin levels [55]. An RCT that included 64 age and gender-matched healthy asymptomatic individuals evaluated levels of IL-1 and TNF-α before, 20 min and 2 h after either a thoracic manipulation that resulted in a cavitation, a thrust to the thoracic spine that did not cause a cavitation (sham SMT), or a control group that received venipuncture. It was found that over the evaluation period, the sham and control individuals showed an increase in their TNF-α and IL-1 concentrations and the group that received manipulations with cavitation showed a decrease [19]. As TNF-α and IL-1 are both strong pro-inflammatory cytokines [78], the findings of this study indicate that the central anti-inflammatory mechanism is influenced following an HVLA controlled vertebral thrust.

The influence of spinal manipulation on central anti-inflammatory mechanisms is further supported by two RCTs that measured the levels of IL-2 in healthy individuals [20,21]. In both of these studies, the IL-2 levels were measured before, 20 min and 2 h after either a thoracic manipulation that resulted in cavitation, a thrust to the thoracic spine that did not cause a cavitation (sham SMT), or a control group that received venipuncture [20]. Teodorczyk-Injeyan and Injeyan [20] found that in vitro synthesis of IL-2 levels increased over the evaluation period irrespective of whether the groups received the adjustment with or without cavitation, but they did not increase in the control group. As the mechanical effects of spinal manipulation may not be directly related to cavitation [80,81,82], the findings of this study indicate that HVLA controlled vertebral thrusts may influence IL-2-regulated biological responses under certain physiological conditions. Teodorczyk-Injeyan and McGregor [21] found that over the evaluation period, IL-2 -induced immunoglobulin G and immunoglobulin M was significantly increased in cultures from individuals who received manipulation with cavitation. Another study found that IL-6 and C-reactive protein (CRP) decreased after HVLA controlled vertebral thrusts with an Activator IV hand-help adjusting device [24]. In contrast, there were no significant changes in concentration of IL-1β, IL-6, IL-8, or IL-10 after 12 weeks of osteopathic treatment in people with non-specific chronic low back pain [26]. However, concentrations of TNF-α reduced significantly in people who received the osteopathic treatment as compared to those who received sham treatment [26]. Similarly, a significant decrease in circulating levels of TNF-α after 12 weeks of osteopathic manual treatment was noted in people with diabetes mellitus and comorbid chronic low back pain [27]. No significant effect of manual treatment on IL-1b, IL-6, TNF-α, and CRP was also noted one and 24 h after the treatment [31]. 

IL-2 is produced by T helper Lymphocytes, called CD4+ cells [79]. IL-2 stimulates the cell-mediated immune response, and has an anti-inflammatory effect by reducing the pro-inflammatory effects of IL-6 [83]. IL-2 controls the growth and differentiation of both T and B Lymphocytes [79]. It also promotes self-tolerance and inhibits the growth of some human tumor cells [79]. IL-2 is currently being used to treat auto-immune disorders and various cancers [84,85,86]. However, there are still unanswered questions about whether this increase has a clinically relevant impact on immunity, whether a similar increase can be seen in people with various pathological conditions, how long this effect lasts, and whether it has a positive impact in patients with autoimmune conditions, or cancer patients, or whether it has an anti-inflammatory effect. More research is needed to answer these questions.

There is some evidence that the impact of HVLA controlled vertebral thrusts on immune mediators may be useful in various pathological conditions. Six sessions of HVLA controlled vertebral thrusts to the affected areas was reported to reduce the elevated inflammatory mediators found in people with low back pain [22]. However, levels did not reduce to the level of the asymptomatic control individuals. People with non-specific, acute and chronic low back pain have elevated levels of some inflammatory mediators compared to control subjects [22,56]. This may be due to the inflammation occurring at the level of joint dysfunction or CSMC problem as micro-trauma may be occurring to the tissues at the site, since the CNS is less accurately aware of what is happening due to the neurological inhibition of the deep small paraspinal muscles. The motor control of the spine at this area is less likely to be appropriate and hence micro-trauma may be occurring repetitively. It has also been suggested that patients with chronic pain, such as chronic low back pain or fibromyalgia, have dysfunctional cortisol levels, which may be the cause of the inflammation and are linked to pain hypersensitivity [56,87]. This makes sense, as cortisol is an immunomodulator and responsible for ensuring that inflammation is not carried on unchecked, which is what is occurring in chronic pain conditions [50,87]. Research suggests that HVLA controlled vertebral thrusts influence cortisol levels, as discussed above, this may provide some insight into pain reduction in these groups.

A study by Selano and Hightower [17] monitored CD4 cell counts in 10 HIV positive individuals over a 6-month period. Five patients received upper cervical only adjustments (treatment group) and five were in a control group. In the adjustment group, 48% higher CD4 counts were noted at the end of the 6-month period, while the control group had an 8% decrease in their levels [17]. A note of caution is due here since the results were not statistically significant. In addition, as the results were poorly reported, it may be heavily swayed by a single person or other limitations of the study. 

Other studies have investigated the impact of HVLA controlled vertebral thrusts on people with other immunologically mediated conditions like asthma [88,89,90,91]. Generally, the results of these studies have not been strong enough to make any conclusions about the effectiveness of HVLA controlled vertebral thrusts to help people with these conditions. Systematic reviews tend to report that either there is not enough evidence to say HVLA controlled vertebral thrusts help children with asthma [90], or that the results are negative [89,92]. Larger, good quality studies are needed to establish whether there is a link between HVLA controlled vertebral thrusts and the immune response in immunologically mediated conditions.

#### 3.1.2. Effects of HVLA Controlled Vertebral Thrust on the Autonomic Nervous System

It is now accepted that the ANS and HPA axis influence the immune response and stress response [70,93,94,95,96,97,98,99,100,101,102,103,104,105,106,107,108,109,110,111,112]. However, these systems are impossible to separate, as they are highly coordinated and physically interconnected [113]. The evidence for an effect of HVLA controlled vertebral thrusts on ANS function related to the immune response is limited [55]. A single, low-quality study found no effect of HVLA controlled vertebral thrusts on epinephrine and norepinephrine levels [54]. Another study found that application of HVLA controlled vertebral thrusts did not influence the HPA axis in symptomatic and asymptomatic males [50]. Most of the literature in this area has focussed on other aspects of ANS function that may be impacted by HVLA controlled vertebral thrusts [114,115]. Some studies have shown that HVLA controlled vertebral thrusts may positively impact various aspects of ANS function, such as heart rate variability, cardiovascular function, respiratory function, gastrointestinal function, and genitourinary function [114,115]. However, most of these studies are basic science studies [114,115] and more clinically relevant trials are needed to evaluate how HVLA controlled vertebral thrusts impact ANS function and whether this is clinically relevant with respect to immune function.

### 3.2. Potential Mechanisms That Link HVLA Controlled Vertebral Thrusts with Immune Function

In the past, it was thought that the CNS and the immune system functioned relatively independently [116]. However, more recently, the strong, interconnected relationship between the CNS and the immune system has received significant attention from the research community [117]. As HVLA controlled vertebral thrusts have been shown to impact the CNS, and the CNS is known to strongly influence immune function, this provides a plausible biological mechanism to explain how HVLA controlled vertebral thrusts could impact immunity. This section will discuss these potential functional links. 

#### 3.2.1. The Link between HVLA Controlled Vertebral Thrust and the Central Nervous System 

A growing body of research conducted by chiropractors has shown that chiropractic HVLA controlled vertebral thrusts (also called chiropractic adjustments) affect the CNS [118,119,120,121,122,123,124,125,126,127,128,129,130,131,132,133,134,135]. It has been hypothesized that adjusting CSMC problems in the spine, alters the afferent input from the small paraspinal muscles around the spine and skull to the brain [123,136]. This affects how the CNS processes and integrates all subsequent sensory input, such that the brain senses more accurately what is happening in and around the body, so there is improved brain–body awareness, adaptability, and coordination and therefore, better function [123,131]. 

One of the keys to this proposed model is the proprioceptive role played by the small intervertebral muscles closest to the spine and skull. It is now well established that these small paraspinal muscles are very important sensors used by the brain to help control posture and spinal movement patterns [137,138]. It has been hypothesized that CSMC problems alter the afferent input from the small paraspinal muscles to the CNS, which impacts the brain’s awareness of what is happening in the body [123,136,139,140,141]. This, in turn, may affect subsequent afferent feedback from associated structures in the spine and limbs, which has a flow-on effect on how the brain processes and interprets other internal and external sensory information. Ultimately, this can lead to altered sensorimotor and multimodal integration and a lack of awareness in the CNS of the state of the body’s internal and external environment [132,136]. Over time, maladaptive changes in neural function affect the way we respond to our internal and external environment, potentially leading to a lack of adaptability and resilience and the development of symptoms, diseases, and disorders [123,136,139,140,141]. If, by adjusting CSMC problems, the associated rapid stretch of the deep paraspinal muscles enhances proprioceptive afferent input from these muscles, this is thought to improve brain–body awareness, adaptability, and resilience [123,127,140,141].

There is a growing body of research that supports this neuroplasticity model of spinal dysfunction and HVLA controlled vertebral thrusts. Some studies have shown neural plastic changes, such as altered sensorimotor integration and motor control, after HVLA controlled vertebral thrusts [123,124,125,128,131,135]. Other studies have shown improvements after HVLA controlled vertebral thrusts in muscle strength and motor neuron excitability in normal healthy adults and elite athletes, as well as in adults with brain injuries, in particular stroke survivors [120,133,135]. Studies have also shown that HVLA controlled vertebral thusts affect cerebellar function, particularly the communication between the cerebellum and the motor cortex, which has a key role in sensorimotor integration [119,121,142]. Thus, it is quite clear that HVLA controlled vertebral thrusts can impact vital parts of the brain involved in creating internal and external schemas or maps of the internal and external environment. 

More importantly, there is evidence suggesting that chiropractic HVLA controlled vertebral thrusts have a significant effect on the prefrontal cortex [134]. Using brain electrical source localization analysis, it has been identified that adjusting CSMC problems in the spine impacts the prefrontal cortex [134]. The prefrontal cortex is a high level processing centre of the CNS and is described as the executive controller of the CNS [134,143]. It is also a key multimodal association cortex and has functions in motor control and sensorimotor integration through directing and sensing perception of self, attention, memory sequence processing, and motivation [143]. The prefrontal cortex is also known to be vital for proper immune system function [94,144,145,146,147,148,149,150,151,152,153,154]. The prefrontal cortex regulates the ANS, directly activates the vagal cholinergic anti-inflammatory system, and impacts the hormonal system, in particular the hypothalamus–pituitary–adrenal (HPA) axis [94,144,145,146,147,148,149,150,151,152,153,154]. In addition, these mechanisms impact pain processing [149,155,156,157] and all aspects of emotional control and mental health [158,159,160,161,162,163]. Therefore, since HVLA controlled vertebral thrusts influence the CNS, in particular the prefrontal cortex, which in turn has an impact on the ANS, HPA axis, and immune system, this provides a biologically plausible mechanism for how HVLA controlled vertebral thrusts may impact immune function. 

#### 3.2.2. The Link between the Central Nervous System and the Immune System 

The CNS and the immune system are the two primary systems in humans and animals that are responsible for detecting and responding rapidly to our internal and external environment, and they are closely linked, both physically and physiologically [164,165]. This bidirectional communication between the two systems helps to maintain homeostasis, where the immune system acts as a sensory system providing information to the CNS and the CNS responds via the neuroendocrine system, autonomic nervous system, and the meningeal lymphatic system [116,164,165,166,167,168,169]. Mounting evidence suggests small clusters of neurons, glial cells, and immune cells, called neuro–immune cell units (NICUs) are found throughout the body and functionally interact with each other to sense what is going on in and around us, to protect us from invading pathogens and injuries [165]. The immune cells are equipped to respond to neurotransmitters and neuropeptides secreted by neuronal signals and neurons and glial cells can sense and respond to immune-derived cytokines [165].

There is emerging evidence showing that the CNS and the immune system are interconnected and function in close association with each other [170,171,172]. Activation of the immune system has been shown to influence brain function, for instance, immune cells and cytokines released in response to peripheral inflammation affect brain processes such as sleep [173,174], appetite and satiety [175,176], learning and memory [177], behavior [178,179], and neurocognitive function [180,181]. The brain is also capable of impacting immunological reactions. Even one’s mental and emotional state can alter the response to disease or a therapeutic intervention [182,183]. For instance, anticipating negative consequences can result in negative outcomes, referred to as nocebo response [182], whereas expecting positive results from an intervention can lead to improvement in disease state as observed in the placebo effect [183]. Moreover, the brain keeps a check on immune activity following a neurological lesion such as stroke, in order to protect itself from an excessive immune response [170,184]. However, this attenuation of immune response makes post-stroke individuals more prone to chest infections [185]. Notably, the side of stroke lesion impacts the immune response, indicating that the left or right hemispheres of the brain may have differential effects on peripheral immune responses [186,187,188,189,190]. This may be due to their distinct connections with the sympathetic nervous system (SNS) and the parasympathetic nervous system (PNS) [191,192,193,194]. Multiple disciplines, including neuroscience, psychology, immunology, and endocrinology, have explored theories of possible neuroimmune mechanisms in diseases, such as neurocognitive disorders like Alzheimer’s disease; respiratory diseases, such as asthma; autoimmune disorders; mood disorders, such as depression; and in general infection and inflammation processes [67,68,69,70,93,116,117,164,166,180,181,195,196,197,198,199,200,201]. Through this research, it is now well established that there is indeed a strong neuro-immune link [116,165,166].

Neuro-immune communication also occurs within the brain tissue through parenchymal resident cells called microglia and immune cells present in the meninges and choroid plexus [170]. In addition, neuro-immune responses also take place at sites where blood vessels directly or indirectly interact with the brain, such as at the circumventricular organs (CVOs) or blood–brain barrier (BBB). As all the above structures are innervated, neuronal activity can influence the immune response by altering the permeability of the BBB, modulating the activity of the epithelial cells in the CVOs, or affecting the secretion of chemotactic substances in the meninges and choroid plexus. For instance, depressive disorders, sleep deprivation, or severe stress reduce BBB permeability [202] and alter B-cell homing [203] or leukocyte trafficking through the choroid plexus [204]. Furthermore, the microglia and other brain cells are also affected by neuronal activity, as the activation of their neurotransmitter receptors affects their function [205,206,207,208,209], indicating complex neuroimmune interactions.

The basis of the neuroimmune dialogue and the reasons for it have been discussed in a recent review [170]. Schiller and Ben-Shaanan [170] argue that the unique features and functions of the brain can provide three additional benefits to the immune system. First, the brain’s ability to integrate and synchronize physiological processes, such as body temperature, blood flow, metabolism, digestion, cardiovascular, and renal function, may be useful to prepare the individual for an upcoming threat or challenge. One example of this is the hypothalamus-mediated loss of appetite, which facilitates fasting behavior to survive a septic shock, as increasing caloric intake is not always useful to support immune activity [210,211]. Immune activation is metabolically costly [212,213,214]; therefore, synchronizing immunity with physiological functions may be useful for an effective immune response. The suprachiasmatic nucleus of the hypothalamus, known as the inner clock, regulates the circadian rhythm, which has been shown to synchronize the immune system [215,216,217]. Another critical structure involved in immune synchronization is the insula. Insula activity is important for detecting proprioceptive inputs and producing corrective actions, that may include immune activity, to promote homeostasis [218,219]. Second, the brain’s ability to make predictions and anticipate threats may be useful to detect and assess danger before it physically harms the individual. The insular cortex, amygdala, and ventromedial nucleus of the hypothalamus are regulators of immune conditioning [220], a response similar to Pavlonian conditioning [221]. Activation of brain areas involved with anticipation of behaviour may lead to immune priming. Priming of antibacterial immunity has been reported with the activation of the ventral tegmental area, which is involved in positive expectations [222,223,224,225]. Pain may be another factor which is important for the prediction of upcoming threats. Pain-sensing neurons and brain areas responsible for modulation of pain, particularly the periaqueductal grey, have been shown to affect immunity [169,226,227,228,229,230]. The primary and secondary somatosensory cortex, the anterior cingulate cortex, the insula, the prefrontal cortex, the thalamus, and the cerebellum are the brain areas most activated by painful stimuli [231]. Lastly, the brain’s capacity to respond quickly, may facilitate rapid immune responses during stressful events, in order to prepare for impending danger [117,232,233]. 

There are two main pathways involved in CNS–immune system communication: (1) the neuroendocrine system and (2) the ANS, including both (a) the SNS and (b) the PNS [67,68,70,94,116,117,164,234]. Other pathways that contribute towards the neuro-immune communication are the sensory system and the meningeal lymphatic system [170]. 

#### 3.2.3. The Neuroendocrine System

The main brain structure that controls the endocrine system is the hypothalamus. It responds to peripheral inflammation by releasing hormones into circulation at the CVO [235,236]. Any change in hypothalamic activity can impact the immune response. For example, injury to the lateral hypothalamus disrupts the number of immune cells in circulation [237,238] and impacts natural killer cell cytotoxicity in the bloodstream [239]. The hypothalamus regulates the endocrine system through the hypothalamic–neurohypophyseal system and the hypothalamic–hypophyseal portal system, which are necessary for effective immune activity [170]. The hypothalamic–neurohypophyseal system, comprising of the hypothalamus and the posterior pituitary gland, directly releases oxytocin [240,241], and arginine–vasopressin (AVP) [242] from the neurosecretory cells into the bloodstream for the regulation of immune activity. The hypothalamic–hypophyseal portal system constitutes the release of hypothalamic hormones, which stimulates the secretion of anterior pituitary hormones into the bloodstream so that it can ultimately cause the release of relevant hormones from the target organ for the regulation of various physiological processes [170]. For instance, the release of cortisol from the adrenal glands regulates the stress response (HPA axis), the release of thyroid hormone from thyroid glands regulates metabolism (hypothalamic–pituitary–thyroid axis), the release of sex hormones from gonads regulates reproduction (hypothalamic–pituitary–gonadal axis), the release of prolactin affects the production of milk in females (hypothalamic–pituitary–prolactin axis) and the release of growth hormones and insulin-like growth factor 1 (IGF-1) regulates growth or blood glucose levels (hypothalamic–pituitary–somatotropic axis). All these hormones impact our immune responses; for instance, estrogen enhances immunity, which is why females have been shown to produce a higher number of antibodies and are less prone to viral infections [243,244]. However, this also makes them more susceptible to autoimmune diseases [245,246]. Another example is thyroid hormone, which activates the proliferation of lymphocytes [247]. Absence (thyroidectomy) or attenuation of this hormone (hypothyroidism) suppresses the immune response [248] or increases the susceptibility to infections [249,250], respectively.

#### 3.2.4. HPA Axis

The hypothalamus, pituitary, and adrenal gland form the HPA axis. The HPA axis can modulate the immune system by responding to immune mediators, such as cytokines and eicosanoids that have been released from the immune cells due to some infectious pathogen, injury, trauma, stress, or any kind of immune challenge [67,68,70,71,93,95,201]. NICU’s detect immune challenges throughout the body and promote the release of cytokines, such as IL-1, IL-6, and TNF-α, and eicosanoids in order to activate a local inflammatory response [67,71,93,201]. Once these immune mediators are released, this is communicated to the brain via blood and vagal sensory afferent fibres [95]. Afferent fibres of the vagus nerve project to the brainstem and then through an ascending link to the hypothalamus [67,68,71,93,95,201]. These immune mediators can then activate the HPA axis. In response, the hypothalamus releases corticotrophin releasing hormone (CRH) which triggers the release of adreno-corticotrophic hormone (ACTH) by the pituitary gland, which in turn stimulates the release of glucocorticoids by the adrenal gland [67,68,70,71,93,201]. Glucocorticoid steroids act in an immunomodulatory manner by regulating and suppressing the immune response through the modulation of cytokines and other inflammatory mediators, in order to limit overactive or detrimental responses [67,68,71,93,95,201]. In a healthy system, this is balanced by other pituitary hormones, such as prolactin, growth hormone and also thyroid hormones, so that chronic immunosuppression does not occur and homeostasis is maintained [196].

#### 3.2.5. The ANS

The other primary mechanism by which the CNS and immune system interacts, is through the ANS, via both the SNS and the PNS divisions [70,93,94,95,96,97]. The dorsal motor vagal nucleus [251] or the rostral ventrolateral medulla [252], locus coeruleus (LC) [253], A5 [254,255], and the rostral raphe pallidus [256,257] are the key areas involved in the regulation of the ANS pathways, which send signals to the periphery and impact immune responses. 

#### 3.2.6. The SNS

The SNS has a dual role in the immune response, as it has both pro-inflammatory and anti-inflammatory actions [95]. The SNS innervates lymphoid organs, such as the spleen, bone marrow, and tonsils, and communicates with immune cells in these organs via the neurotransmitter norepinephrine, also known as noradrenaline [70,93,95,96,97]. The SNS can modulate blood flow and immune cell distribution [165]. Through the SNS’s release of norepinephrine, it acts to modulate the response of CD4 T-cells and B-cells during an immune response against an antigen [166].

The SNS also interacts with the adrenal gland through adrenoreceptors, here they act to stimulate glucocorticoid (cortisol and cortisone) and catecholamine (epinephrine and norepinephrine or adrenaline and noradrenaline) release from the adrenal glands, which then modulate the level of cytokines. The SNS can, therefore, also have a direct hormonal effect by activating the adrenal glands. 

#### 3.2.7. The PNS

The PNS also has a known neuro-immune effect via the vagus nerve [94,95]. This is known as the cholinergic anti-inflammatory system, because the vagus nerve releases acetylcholine (ACH). The ACH release, due to parasympathetic activity, inhibits pro-inflammatory cytokines such as IL-6 [83,94]. The activity of the PNS drives this cholinergic anti-inflammatory system by the release of ACH in the reticuloendothelial system, which includes the liver, heart, spleen, lungs, blood, general connective tissue, gastrointestinal tract, bone marrow, and lymph nodes. The ACH interacts with receptors on macrophages to inhibit the release of pro-inflammatory cytokines. This is how the PNS can modulate the function of the immune system, but it should be noted that the PNS also plays an important sensory role in the body/brain connection, as it senses the presence of pro-inflammatory cytokines in the body and conveys these signals to the brain.

#### 3.2.8. The Sensory System and the Meningeal Lymphatic System

The sensory system, via sensory neurons, detects potential threats in peripheral tissue and releases neuropeptides that influence the immune cells in the periphery [258,259]. They also send relevant information from the periphery to the brain [170]. An additional way by which the brain influences the immune function is through the meningeal lymphatic system. The brain releases brain-specific antigens and immune cells into the lymphatic vessels surrounding the brain, which transports it to the peripheral lymph nodes to affect the peripheral and central immune response [170].

#### 3.2.9. Stress Negatively Impacts Immune Function and Vertebral Motor Control

It is now accepted that negative emotional stress has a major impact on the CNS and immune function [69,144,151,195,260,261,262]. Stressful mental and emotional states suppress peripheral immunological activity and cause increased susceptibility to infection, as noted in depression [263,264,265,266]. In addition to infections, injuries, pathogens or inflammation, emotional stress can also activate our ANS, in particular the SNS and it can activate the HPA stress axis in a way that has negative long term implications for our health [69,151,195,196,260,267,268]. This increases levels of glucocorticoids (like cortisol) and catecholamines (like epinephrine and norepinephrine) that can alter cytokine levels (such as increasing IL-6 pro-inflammatory cytokines), which then alters the levels of inflammation throughout the body [67,68,69,70,96,97,116,117,164,195,268]. Over time, this can weaken the immune system, and put the individual at risk of having more frequent, prolonged, or excessive immune reactions [67,68,69,70,96,97,116,117,164,195,268]. We also know negative emotional stress inhibits the prefrontal cortex from operating properly [144,260], which also has detrimental effects on the ability of the PNS to activate the cholinergic anti-inflammatory pathway [94,144,145]. Dysfunction within the prefrontal cortex is also likely to impact on its normal inhibition of the HPA axis [154]. Therefore, with stress increasing inflammation via the HPA axis and the SNS, and supressing prefrontal cortex function, which reduces its suppression of the HPA axis and inhibits its activation of the PSN’s cholinergic anti-inflammatory system (via the vagal nerve), it is not surprising that negative emotional stress, over long periods of time, may lead to higher inflammatory levels in the body [269,270,271].

Excessive levels of inflammation in adults has been linked to a host of mental and physical diseases and disorders, such as autoimmune diseases, diabetes, coronary artery disease, cancer, Alzheimer’s disease, obesity, depression, anxiety, PTSD, pulmonary disease, various neurological diseases, chronic pain and arthritis, ulcerative colitis, and Crohn’s disease [95,98,99,100,101,102,103,104,105,106,107,108,109,110,111,112]. Chronic inflammation has even been suggested to be one of the key biological mechanisms that leads to a decline in physical function, potentially then, as we age, resulting in frailty, disability, and ultimately, death [98,272]. Acute and chronic stress have a distinct impact on the immune response. Studies on animals and humans have shown that acute stress enhances the immune response [232,273], whereas chronic stress suppresses the immune system [274,275,276]. For example, acute stress experienced during parachute jumping or solving a difficult arithmetic exam increases the activity and number of natural killer cells and CD8^+^ T cells [233,277]. Prolonged exposure to a stressful life situation has been shown to attenuate immune gene cell expression [169,278,279,280]. Research studies have established that chronic stress and psychological stress slow wound healing, can increase the severity and duration of infectious diseases, such as respiratory infections, and it can reactivate latent viruses in the body, and there is even evidence that suggests that stress can reduce the response to some vaccines [69,93,195,196]. Other studies have shown that people who experience higher levels of psychological stress are more susceptible to getting the common cold, immune-related disorders, and respiratory infections [268,281]. Chronic stress causes accumulation of pro-inflammatory leukocytes due to hematopoietic stem cell proliferation in the bone marrow [282].

Interestingly, there is growing evidence that shows that stress may also impact vertebral motor control [283,284,285]. Executing tasks under the influence of psychological stress has been shown to alter muscle activation patterns, as noted in trunk muscles during a lifting task [286] and shoulder muscles during a keyboard task [287]. Stress may also have differential effects on muscle activation, depending on the type of muscle. Exposure to acute psychological stress increases the activation of bigger muscle groups while inhibiting the deep small muscles [288]. Increased activation in the upper trapezius muscles has been noted during acute psychological stress [289]. Stress-induced increase in SNS activation or disinhibition of the reticular formation, which under non-stressful situations is responsible for inhibiting the trapezius muscle during ongoing movement, has been suggested to cause the increased trapezius activation [290]. On the other hand, acute psychological stress has also been shown to inhibit small paraspinal muscles close to the spine and the skull [288]. Both these responses can be explained by the stress-induced effects of the SNS on muscle spindle activity, such that increased sympathetic outflow depresses muscle spindle activation and changes the feedback control of muscle length and movement [291]. An example of this is seen when enhanced sympathetic outflow attenuates precise and fine movement control to ensure fast and gross movements during a life-threatening situation. However, if the increase in sympathetic outflow occurs in situations that require accurate and continuous proprioceptive feedback, it may result in inaccurate performance outputs that result in maladaptation. Such stress-induced changes in muscle afferent signaling that lead to maladaptations may be responsible for chronic muscle pain and dysfunction [291,292]. 

Another likely reason for stress-induced change in vertebral motor control may be related to pain or pain-related fear of movement. In people with chronic pain, alterations in paraspinal muscle control have been found to be associated with self-reported measures of anxiety and distress, instead of just the intensity of pain [293,294,295]. This may indicate that the stress associated with pain may change the activation of trunk muscles. A delay in the response of deep spinal muscles during fast arm movements has been noted when people with experimentally induced pain performed tasks with negative feedback of performance and negative cues [283]. There is also evidence of differential trunk muscle activity in fearful and non-fearful back pain patients, such that people who are more fearful have less relaxation [296] and lower endurance of paraspinal muscles at the end range of trunk flexion [297]. Another study found that fear of movement resulted in decreased upper trapezius muscle activity in people with post-traumatic neck pain and that the effect was stronger when the intensity of pain was higher [298]. A positive link between fear of movement and trunk stiffness was reported in people with low back pain, with greater fear of movement and fear-avoidance beliefs being associated with greater trunk stiffness during forward perturbation [299].Therefore, stress related to pain or negative emotions alters the muscle activation patterns of our body and impacts vertebral motor control. This occurs by stress-induced effects of SNS on muscle spindle activity that changes the muscle activation and cause maladaptation. If the CNS is not able to perceive accurately what is happening at that spinal level, with the small deep paraspinal muscles neurologically inhibited, then it makes logical sense that it cannot control this part of the spine appropriately, hence a central segmental motor control problem may arise that over time leads to repeated microtrauma at that area of the spine, which chiropractors and other manual therapists can palpate. Therefore, stress may be a major cause of what chiropractors refers to as vertebral subluxations, i.e., “a self-perpetuating, central segmental motor control problem that involves a joint, such as a vertebral motion segment, that is not moving appropriately, resulting in ongoing maladaptive neural plastic changes that interfere with the central nervous system’s ability to self-regulate, self-organize, adapt, repair and heal” [300].

#### 3.2.10. Summary of the Neuroimmune Link

Stress weakens our immune response and makes us more susceptible to infections such as respiratory diseases [95,98,99,100,101,102,103,104,105,106,107,108,109,110,111,112]. Attenuation of the immune response occurs due to increased inflammation induced by stress either directly through the activation of SNS and HPA axis or indirectly via suppression of the prefrontal cortex to reduce the inhibition of HPA axis and supress anti-inflammatory PNS activity. Furthermore, under normal circumstances, the neuro-immune dialogue is integral for the maintenance of homeostasis in the body. There is substantial evidence that shows the nervous system, the hormonal system and the immune system communicate with one another and are intimately linked in their functions [70,93,94,95,96,97]. The NICUs present within the brain and throughout the body forms the basis of this neuro-immune interaction such that the neurons and glial cells sense and respond to immune-derived cytokines, while the immune cells are equipped to respond to neurotransmitters and neuropeptides secreted by neuronal signals [165]. This interaction allows the immune system to maximise its functioning by capitalizing on the brains ability to synchronize physiological processes, anticipate threats and act swiftly. Various brain structures, including the suprachiasmatic nucleus of the hypothalamus, the amygdala, the ventromedial nucleus of the hypothalamus, the periaqueductal grey, and the insular cortex impact the immune system [170,215,216,220,229]. More importantly, the prefrontal cortex is critically involved in regulating the autonomic nervous system, the endocrine system and therefore the immune system [94,144,145,146,147,148,149,150,151,152,153,154]. 

## 4. Implications, limitations, and Recommendations for Future Research

It is clearly now well-established that the CNS and immune system are very closely linked and rely on each other to accurately detect an immune challenge, and together they launch and regulate an appropriate immune response [70,93,94,95,96,97]. The NICUs around the body sense invading pathogens, stress, injury, or any other immune challenge. This is signaled to the brain in two ways: directly via vagus afferent nerve fibers, or indirectly via the bloodstream. The afferent nerve fibers, particularly the sensory neurons, not only carry the information from the periphery to the brain but also release neuropeptides in the periphery to impact the local immune response [258,259]. Immune-related signals carrying information about the brain’s condition is delivered from the brain to the peripheral lymph nodes via the meningeal lymphatic system. The brain mainly modulates the immune system via two main pathways, the HPA axis and ANS. The HPA axis and both divisions of the ANS are activated by immunogenic stimuli and both contribute to the modulation of inflammation. They do this by releasing glucocorticoids (cortisol and cortisone) and catecholamines (epinephrine and norepinephrine), that regulate inflammatory mediators such as cytokines. The prefrontal cortex is involved in regulating the autonomic nervous system, the endocrine system and the immune system [154]. Additional communication between the CNS and the immune system occurs within the brain parenchyma and sites such as the BBB or CVO. The CNS and immune system function is affected by stress. Stress affects our immune response and makes us more prone to infections [95,98,99,100,101,102,103,104,105,106,107,108,109,110,111,112]. Stress, pain, or pain-related stressors also impact our muscle activation patterns during movement, which may lead to maladaptive changes over time. 

**Figure 1:**
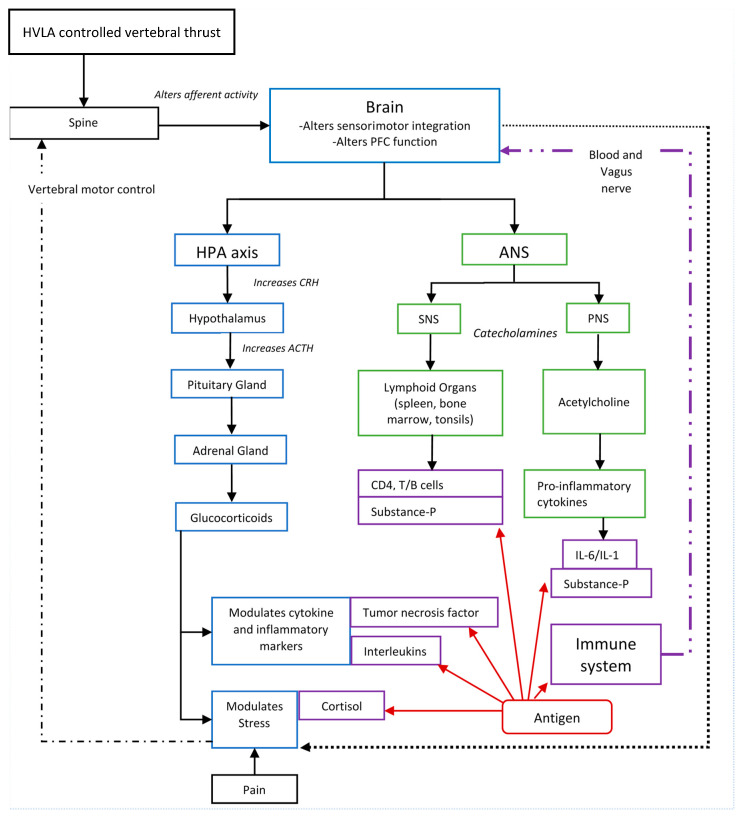
Illustration demonstrating the HVLA controlled vertebral thrust–immune system link. PFC: Prefrontal cortex, HPA: Hypothalamus–pituitary–adrenal axis, ANS: Autonomic nervous system, SNS: Sympathetic nervous system, PNS: Parasympathetic nervous system, CRH: Corticotrophin releasing hormone, ACTH: Adreno–corticotropic hormone, IL: Interleukin.

A growing body of research suggests HVLA thrusts directed at CSMC problems affect the CNS [118,119,120,121,122,123,124,126,127,128,129,130,131,132,133,134,135], and in particular affect processing in the prefrontal cortex [134]. This provides a biologically plausible mechanism for how HVLA controlled vertebral thrusts could have an impact on immune function [55]. Figure 1 depicts many of the potential links between HVLA controlled vertebral thrusts and immune function previously discussed.

This review of the literature shows that there is basic science evidence that HVLA controlled vertebral thrusts can modulate immune mediators, such as neurotensin, oxytocin, substance-P, and interleukin levels in healthy asymptomatic individuals in the short-term [55]. In addition, they may influence cortisol levels in symptomatic and asymptomatic individuals [29]. However, most of the studies included in this review evaluated the immune mediators immediately pre- and post-HVLA controlled vertebral thrusts, or a few hours later on the same day [15,20,21,28,29]. Therefore, it is not known for how long these changes in levels of immune mediators last. The studies did not evaluate what these changes mean in patient populations in the long-term or whether the changes are clinically relevant. Therefore, although the current basic science research on this topic is positive, one needs to be cautious about making any claims on the use of HVLA controlled vertebral thrusts to enhance a patient’s immunity, because no high quality clinical trials have been performed to assess this important question. 

In the studies that were reviewed, there were also some important differences in the way that the markers were collected. For example, some markers were collected via blood samples and some via saliva. The collection of blood samples, which requires needle puncture, could induce a stress response itself and affect the results. Such limitations must be considered when discussing these results. It is also important to know that results from some studies are conflicting. Therefore, the whole picture is far from clear at this stage. Whether changes in the immune mediators observed after HVLA controlled vertebral thrusts enhances immune function, reduces inflammation, promotes recovery, or suppresses stress-associated immune disorders in symptomatic individuals is not yet known. Chiropractors and other manual therapists should be aware that clinically relevant research has not yet been done that shows that HVLA controlled vertebral thrusts enhance immune function [301]. This is important for all manual therapists to know when promoting the potential benefits of HVLA controlled vertebral thrusts or when sharing information related to HVLA controlled vertebral thrusts and immunity or the COVID-19 pandemic. Further long term studies, with appropriate sample sizes, that investigate immune responses or clinically relevant measures of immune function, are required to obtain more insights about the biological effects of HVLA controlled vertebral thrusts on immune function [56]. Studies such as these are imperative in order to gain a better understanding about the potential impact of HVLA controlled vertebral thrusts on immune function. 

## 5. Conclusions

There is substantial evidence suggesting that the nervous system, the hormonal system and the immune system communicate with one another and are intimately linked in their functions [70,93,94,95,96,97]. This communication is essential for the body’s ability to protect itself and involves a variety of immune mediators, including cytokines, neurotransmitters, hormones, and humoral factors [67,68,70,94,116,117,164,234]. Furthermore, the prefrontal cortex is critically involved in regulating the autonomic nervous system, the HPA axis, and the immune system [94,144,145,146,147,148,149,150,151,152,153,154]. Neuro-immune communication is affected by emotional or pain-related stress [69,144,151,195,260,261,262]. Stress activates the SNS and HPA axis to increase inflammation in the body. Stress also suppresses the prefrontal cortex, which in turn reduces its inhibitory control on the HPA axis and inhibits the anti-inflammatory PNS activity. This stress-induced inflammation weakens the immune response [95,98,99,100,101,102,103,104,105,106,107,108,109,110,111,112]. The stress-induced SNS activity also alters the muscle activation patterns to impair vertebral motor control [283,284,285], thus can cause the establishment of CSMC problems. HVLA controlled vertebral thrusts have been shown to affect vertebral motor control [123,124,125,128,131,135], the prefrontal cortex [134], and the levels of immune mediators in the body that are important for a healthy immune response [15,16,17,18,19,20,21,22,23,24,25,26,27,28,29,30,31,32]. Although there is a biologically plausible mechanism for how HVLA controlled vertebral thrusts may influence the immune system, it is not yet known whether these changes have a clinically relevant impact on immunity [55]. More large-scale clinical trials are needed to understand whether HVLA controlled vertebral thrusts can improve immunity or recovery from illness.

## Data Availability

Not applicable.

## Supplementary Materials

The following are available online at https://www.mdpi.com/article/10.3390/medicina57060536/s1. Supplementary S1: The search strategy; Supplementary S2: The effect of high-velocity-low-amplitude controlled vertebral thrusts on immune and endocrine markers. TNF: tumour necrosis factor, IL: interleukin, CRP: C-reactive protein. Shaded rows show publications with no reported changes after high-velocity, low-amplitude vertebral thrusts.

## References

[1] Ranasinghe C., Ozemek C., Arena R. (2020). Exercise and well-being during COVID 19-Time to boost your immunity. Expert Rev. Anti Infect. Ther..

[2] Wortham J.M., Lee J.T., Althomsons S., Latash J., Davidson A., Guerra K. (2020). Characteristics of Persons Who Died with COVID-19—United States, February 12–May 18, 2020. MMWR Morb. Mortal. Wkly. Rep..

[3] Lai Q., Spoletini G., Bianco G., Graceffa D., Agnes S., Rossi M., Lerut J. (2020). SARS-CoV2 and immunosuppression: A double-edged sword. Transpl. Infect. Dis..

[4] Sadia A., Basra M.A.R. (2020). Therapeutic dilemma in the repression of severe acute respiratory syndrome coronavirus-2 proteome. Drug Dev. Res..

[5] Florindo H.F., Kleiner R., Vaskovich-Koubi D., Acúrcio R.C., Carreira B., Yeini E., Tiram G., Liubomirski Y., Satchi-Fainaro R. (2020). Immune-mediated approaches against COVID-19. Nat. Nanotechnol..

[6] De Geest B., Ye T., Zhong Z., García-Sastre A., Schotsaert M. (2020). Current status of COVID-19 (pre)clinical vaccine development. Angew Chem. Int. Ed. Engl..

[7] Gao Z., Lee J.E., McDonough D.J., Albers C. (2020). Virtual Reality Exercise as a Coping Strategy for Health and Wellness Promotion in Older Adults during the COVID-19 Pandemic. J. Clin. Med..

[8] Dicker D., Bettini S., Farpour-Lambert N., Frühbeck G., Golan R., Goossens G., Halford J., O’Malley G., Mullerova D., Ramos Salas X. (2020). Obesity and COVID-19: The Two Sides of the Coin. Obes. Facts.

[9] De Faria Coelho-Ravagnani C., Corgosinho F.C., Sanches F.F.Z., Prado C.M.M., Laviano A., Mota J.F. (2020). Dietary recommendations during the COVID-19 pandemic. Nutr. Rev..

[10] Kwon C.Y., Kwak H.Y., Kim J.W. (2020). Using Mind-Body Modalities via Telemedicine during the COVID-19 Crisis: Cases in the Republic of Korea. Int. J. Environ. Res. Public Health.

[11] El Morr C., Ritvo P., Ahmad F., Moineddin R. (2020). Effectiveness of an Eight-Week Web-Based Mindfulness Virtual Community Intervention for University Students on Symptoms of Stress, Anxiety, and Depression: A Randomized Controlled Trial. JMIR Ment. Health.

[12] Viswanathan R., Myers M.F., Fanous A.H. (2020). Support Groups and Individual Mental Health Care via Video Conferencing for Frontline Clinicians During the COVID-19 Pandemic. Psychosomatics.

[13] Daluiso-King G., Hebron C. (2020). Is the biopsychosocial model in musculoskeletal physiotherapy adequate? An evolutionary concept analysis. Physiother. Theory Pract..

[14] Gliedt J.A., Schneider M.J., Evans M.W., King J., Eubanks J.E. (2017). The biopsychosocial model and chiropractic: A commentary with recommendations for the chiropractic profession. Chiropr. Man. Ther..

[15] Brennan P.C., Kokjohn K., Kaltinger C.J., Lohr G.E., Glendening C., Hondras M.A., McGregor M., Triano J.J. (1991). Enhanced phagocytic cell respiratory burst induced by spinal manipulation: Potential role of substance P. J. Manip. Physiol. Ther..

[16] Brennan P.C., Graham M.A., Triano J.J., Hondras M.A., Anderson R.J. (1994). Lymphocyte profiles in patients with chronic low back pain enrolled in a clinical trial. J. Manip. Physiol. Ther..

[17] Selano J., Hightower B., Pfleger B., Collins K., Grostic J. (1994). The effects of specific upper cervical adjustments on the CD4 counts of HIV positive patients. Chiropr. Res. J..

[18] Davison S., Parkin-Smith G. (2003). The possible effect of upper cervical chiropractic manipulation on short-term lymphocytic response: A pilot study. Eur. J. Chiropr..

[19] Teodorczyk-Injeyan J.A., Injeyan H.S., Ruegg R. (2006). Spinal manipulative therapy reduces inflammatory cytokines but not substance P production in normal subjects. J. Manip. Physiol. Ther..

[20] Teodorczyk-Injeyan J.A., Injeyan H.S., McGregor M., Harris G.M., Ruegg R. (2008). Enhancement of in vitro interleukin-2 production in normal subjects following a single spinal manipulative treatment. Chiropr. Osteopathy.

[21] Teodorczyk-Injeyan J.A., McGregor M., Ruegg R., Injeyan H.S. (2010). Interleukin 2-regulated in vitro antibody production following a single spinal manipulative treatment in normal subjects. Chiropr. Osteopathy.

[22] Teodorczyk-Injeyan J.A., McGregor M., Triano J.J., Injeyan S.H. (2018). Elevated production of nociceptive CC chemokines and sE-selectin in patients with low back pain and the effects of spinal manipulation: A nonrandomized clinical trial. Clin. J. Pain.

[23] Teodorczyk-Injeyan J.A., Triano J.J., Gringmuth R., DeGraauw C., Chow A., Injeyan H.S. (2021). Effects of spinal manipulative therapy on inflammatory mediators in patients with non-specific low back pain: A non-randomized controlled clinical trial. Chiropr. Man. Ther..

[24] Roy R.A., Boucher J.P., Comtois A.S. (2010). Inflammatory response following a short-term course of chiropractic treatment in subjects with and without chronic low back pain. J. Chiropr. Med..

[25] Padayachy K., Vawda G.H.M., Shaik J., McCarthy P.W. (2010). The immediate effect of low back manipulation on serum cortisol levels in adult males with mechanical low back pain. Clin. Chiropr..

[26] Licciardone J.C., Kearns C.M., Hodge L.M., Bergamini M.V. (2012). Associations of cytokine concentrations with key osteopathic lesions and clinical outcomes in patients with nonspecific chronic low back pain: Results from the OSTEOPATHIC Trial. J. Am. Osteopath. Assoc..

[27] Licciardone J.C., Kearns C.M., Hodge L.M., Minotti D.E. (2013). Osteopathic manual treatment in patients with diabetes mellitus and comorbid chronic low back pain: Subgroup results from the OSTEOPATHIC Trial. J. Am. Osteopath. Assoc..

[28] Molina-Ortega F., Lomas-Vega R., Hita-Contreras F., Manzano G.P., Achalandabaso A., Ramos-Morcillo A.J., Martínez-Amat A. (2014). Immediate effects of spinal manipulation on nitric oxide, substance P and pain perception. Man. Ther..

[29] Plaza-Manzano G., Molina F., Lomas-Vega R., Martínez-Amat A., Achalandabaso A., Hita-Contreras F. (2014). Changes in biochemical markers of pain perception and stress response after spinal manipulation. J. Orthop. Sports Phys. Ther..

[30] Sampath K.K., Botnmark E., Mani R., Cotter J.D., Katare R., Munasinghe P.E., Tumilty S. (2017). Neuroendocrine response following a thoracic spinal manipulation in healthy men. J. Orthop. Sports Phys. Ther..

[31] Degenhardt B.F., Johnson J.C., Fossum C., Andicochea C.T., Stuart M.K. (2017). Changes in cytokines, sensory tests, and self-reported pain levels after manual treatment of low back pain. Clin. Spine Surg..

[32] Lohman E.B., Pacheco G.R., Gharibvand L., Daher N., Devore K., Bains G., AlAmeri M., Berk L.S. (2019). The immediate effects of cervical spine manipulation on pain and biochemical markers in females with acute non-specific mechanical neck pain: A randomized clinical trial. J. Man. Manip..

[33] Pickar J.G., Wheeler J.D. (2001). Response of muscle proprioceptors to spinal manipulative-like loads in the anesthetized cat. JMPT.

[34] Sung P.S., Kang Y.M., Pickar J.G. (2005). Effect of spinal manipulation duration on low threshold mechanoreceptors in lumbar paraspinal muscles: A preliminary report. Spine.

[35] Pickar J.G., Kang Y.M. (2006). Paraspinal muscle spindle responses to the duration of a spinal manipulation under force control. J. Manip. Physiol..

[36] Pickar J.G., Sung P.S., Kang Y.M., Ge W. (2007). Response of lumbar paraspinal muscles spindles is greater to spinal manipulative loading compared with slower loading under length control. Spine J..

[37] Cao D., Reed W., Long C., Kawchuk G., Pickar J. (2013). Effects of thrust amplitude and duration of high-velocity, low-amplitude spinal manipulation on lumbar muscle spindle responses to vertebral position and movement. J. Manip. Physiol. Ther..

[38] Brown S.H., Gregory D.E., Carr J.A., Ward S.R., Masuda K., Lieber R.L. (2011). ISSLS prize winner: Adaptations to the multifidus muscle in response to experimentally induced intervertebral disc degeneration. Spine.

[39] Hodges P., Holm A.K., Hansson T., Holm S. (2006). Rapid atrophy of the lumbar multifidus follows experimental disc or nerve root injury. Spine.

[40] Hodges P.W., Galea M.P., Holm S., Holm A.K. (2009). Corticomotor excitability of back muscles is affected by intervertebral disc lesion in pigs. Eur. J. Neurosci..

[41] Hodges P.W., James G., Blomster L., Hall L., Schmid A.B., Shu C., Little C., Melrose J. (2014). Can proinflammatory cytokine gene expression explain multifidus muscle fiber changes after an intervertebral disc lesion?. Spine.

[42] Hodges P.W., James G., Blomster L., Hall L., Schmid A., Shu C., Little C., Melrose J. (2015). Multifidus Muscle Changes After Back Injury Are Characterized by Structural Remodeling of Muscle, Adipose and Connective Tissue, but Not Muscle Atrophy: Molecular and Morphological Evidence. Spine.

[43] James G., Blomster L., Hall L., Schmid A.B., Shu C.C., Little C.B., Melrose J., Hodges P.W. (2016). Mesenchymal Stem Cell Treatment of Intervertebral Disc Lesion Prevents Fatty Infiltration and Fibrosis of the Multifidus Muscle, but not Cytokine and Muscle Fiber Changes. Spine.

[44] Cooley J.R., Walker B.F., Ardakani E.M., Kjaer P., Jensen T.S., Hebert J.J. (2018). Relationships between paraspinal muscle morphology and neurocompressive conditions of the lumbar spine: A systematic review with meta-analysis. BMC Musculoskelet. Disord..

[45] Pedler A., McMahon K., Galloway G., Durbridge G., Sterling M. (2018). Intramuscular fat is present in cervical multifidus but not soleus in patients with chronic whiplash associated disorders. PLoS ONE.

[46] Elliott J.M., Courtney D.M., Rademaker A., Pinto D., Sterling M.M., Parrish T.B. (2015). The rapid and progressive degeneration of the cervical multifidus in whiplash: A MRI study of fatty infiltration. Spine.

[47] Burns E., Chipchase L.S., Schabrun S.M. (2016). Primary sensory and motor cortex function in response to acute muscle pain: A systematic review and meta-analysis. Eur. J. Pain.

[48] Chang W.-J., Buscemi V., Liston M.B., McAuley J.H., Hodges P.W., Schabrun S.M. (2019). Sensorimotor cortical activity in acute low back pain: A cross-sectional study. J. Pain.

[49] Meier M.L., Vrana A., Schweinhardt P. (2018). Low Back Pain: The Potential Contribution of Supraspinal Motor Control and Proprioception. Neuroscientist.

[50] Christian G.F., Stanton G.J., Sissons D., How H.Y., Jamison J., Alder B., Fullerton M., Funder J.W. (1988). Immunoreactive ACTH, beta-endorphin, and cortisol levels in plasma following spinal manipulative therapy. Spine.

[51] Luisetto G., Spano D., Steiner W., Tagliaro F., Darling P., Campacci R. (1982). Plasma Levels of Beta-Endorphin and Calcitonin Before and After Manipulative Treatment of Patients with Cervical Arthrosis and Barre’s Syndrome. Chiropractic Interprofessional Research.

[52] Tuchin P.J. (1998). The effect of chiropractic spinal manipulative therapy on salivary cortisol levels. Australas. Chiropr. Osteopathy.

[53] Whelan T.L., Dishman J.D., Burke J., Levine S., Sciotti V. (2002). The effect of chiropractic manipulation on salivary cortisol levels. J. Manip. Physiol. Ther..

[54] Puhl A.A., Injeyan H.S. (2012). Short-term effects of manipulation to the upper thoracic spine of asymptomatic subjects on plasma concentrations of epinephrine and norepinephrine-a randomized and controlled observational study. J. Manip. Physiol. Ther..

[55] Kovanur-Sampath K., Mani R., Cotter J., Gisselman A.S., Tumilty S. (2017). Changes in biochemical markers following spinal manipulation-a systematic review and meta-analysis. Musculoskelet. Sci. Pract..

[56] Colombi A., Testa M. (2019). The Effects Induced by Spinal Manipulative Therapy on the Immune and Endocrine Systems. Medicina.

[57] Pothoulakis C., Castagliuolo I., Leeman S.E. (1998). Neuroimmune Mechanisms of Intestinal Responses to Stress: Role of Corticotropin-Releasing Factor and Neurotensin. Ann. N. Y. Acad. Sci..

[58] Tyler-McMahon B.M., Boules M., Richelson E. (2000). Neurotensin: Peptide for the next millennium. Regul. Pept..

[59] Kalafatakis K., Triantafyllou K. (2011). Contribution of neurotensin in the immune and neuroendocrine modulation of normal and abnormal enteric function. Regul. Pept..

[60] Da Silva L., Neves B.M., Moura L., Cruz M.T., Carvalho E. (2011). Neurotensin downregulates the pro-inflammatory properties of skin dendritic cells and increases epidermal growth factor expression. Biochim. Biophys. Acta BBA Mol. Cell Res..

[61] Li T., Wang P., Wang S.C., Wang Y.-F. (2017). Approaches mediating oxytocin regulation of the immune system. Front. Immunol..

[62] Insel T.R. (2010). The challenge of translation in social neuroscience: A review of oxytocin, vasopressin, and affiliative behavior. Neuron.

[63] Mashaghi A., Marmalidou A., Tehrani M., Grace P.M., Pothoulakis C., Dana R. (2016). Neuropeptide substance P and the immune response. Cell. Mol. Life Sci..

[64] Johnson M., Young A.D., Marriott I. (2017). The therapeutic potential of targeting substance P/NK-1R interactions in inflammatory CNS disorders. Front. Cell. Neurosci..

[65] Vink R., Gabrielian L., Thornton E. (2017). The role of substance P in secondary pathophysiology after traumatic brain injury. Front. Neurol..

[66] Roviš D., Vasiljev V., Jenko-Pražnikar Z., Petelin A., Drevenšek G., Peruč D., Černelič-Bizjak M. (2021). Mental health and drug use severity: The role of substance P, neuropeptide Y, self-reported childhood history of trauma, parental bonding and current resiliency. J. Ment. Health.

[67] Buckingham J.C., Loxley H.D., Christian H.C., Philip J.G. (1996). Activation of the HPA axis by immune insults: Roles and interactions of cytokines, eicosanoids, and glucocorticoids. Pharmacol. Biochem. Behav..

[68] Chrousos G.P. (1995). The hypothalamic–pituitary–adrenal axis and immune-mediated inflammation. N. Engl. J. Med..

[69] Godbout J.P., Glaser R. (2006). Stress-induced immune dysregulation: Implications for wound healing, infectious disease and cancer. J. Neuroimmune Pharmacol..

[70] Herkenham M., Kigar S.L. (2017). Contributions of the adaptive immune system to mood regulation: Mechanisms and pathways of neuroimmune interactions. Prog. Neuro Psychopharmacol. Biol. Psychiatry.

[71] Mulla A., Buckingham J.C. (1999). Regulation of the hypothalamo–pituitary–adrenal axis by cytokines. Best Pract. Res. Clin. Endocrinol. Metab..

[72] Dedovic K., Duchesne A., Andrews J., Engert V., Pruessner J.C. (2009). The brain and the stress axis: The neural correlates of cortisol regulation in response to stress. Neuroimage.

[73] Blackburn-Munro G., Blackburn-Munro R. (2003). Pain in the brain: Are hormones to blame?. Trends Endocrinol. Metab..

[74] McEwen B.S., Kalia M. (2010). The role of corticosteroids and stress in chronic pain conditions. Metabolism.

[75] Fries E., Hesse J., Hellhammer J., Hellhammer D.H. (2005). A new view on hypocortisolism. Psychoneuroendocrinology.

[76] Heim C., Ehlert U., Hellhammer D.H. (2000). The potential role of hypocortisolism in the pathophysiology of stress-related bodily disorders. Psychoneuroendocrinology.

[77] Sorrells S.F., Caso J.R., Munhoz C.D., Sapolsky R.M. (2009). The stressed CNS: When glucocorticoids aggravate inflammation. Neuron.

[78] Banchereau J., Pascual V., O’garra A. (2012). From IL-2 to IL-37: The expanding spectrum of anti-inflammatory cytokines. Nat. Immunol..

[79] Olejniczak K.A., Kasprzak A.A. (2008). Biological properties of interleukin 2 and its role in pathogenesis of selected diseases—A review. Med. Sci. Monit..

[80] Evans D.W. (2002). Mechanisms and effects of spinal high-velocity, low-amplitude thrust manipulation: Previous theories. J. Manip. Physiol. Ther..

[81] Evans D.W., Breen A.C. (2006). A biomechanical model for mechanically efficient cavitation production during spinal manipulation: Prethrust position and the neutral zone. J. Manip. Physiol. Ther..

[82] Reggars J.W. (1998). The therapeutic benefit of the audible release associated with spinal manipulative therapy: A critical review of the literature. Australas. Chiropr. Osteopathy.

[83] Ross S.H., Cantrell D.A. (2018). Signaling and function of interleukin-2 in T lymphocytes. Annu. Rev. Immunol..

[84] Marcovecchio M.L., Wicker L.S., Dunger D.B., Dutton S.J., Kopijasz S., Scudder C., Todd J.A., Johnson P.R. (2020). Interleukin-2 Therapy of Autoimmunity in Diabetes (ITAD): A phase 2, multicentre, double-blind, randomized, placebo-controlled trial. Wellcome Open Res..

[85] Charych D., Khalili S., Dixit V., Kirk P., Chang T., Langowski J., Rubas W., Doberstein S.K., Eldon M., Hoch U. (2017). Modeling the receptor pharmacology, pharmacokinetics, and pharmacodynamics of NKTR-214, a kinetically-controlled interleukin-2 (IL2) receptor agonist for cancer immunotherapy. PLoS ONE.

[86] Mann T.K., Dail R.B., Bailey D.E. (2016). Cognitive and Affective Symptoms Experienced by Cancer Patients Receiving High-Dose Intravenous Interleukin-2 Therapy: An Integrative Literature Review. Cancer Nurs..

[87] Hannibal K.E., Bishop M.D. (2014). Chronic stress, cortisol dysfunction, and pain: A psychoneuroendocrine rationale for stress management in pain rehabilitation. Phys. Ther..

[88] Balon J., Aker P.D., Crowther E.R., Danielson C., Cox P.G., O’Shaughnessy D., Walker C., Goldsmith C.H., Duku E., Sears M.R. (1998). A Comparison of Active and Simulated Chiropr.actic Manipulation as Adjunctive Treatment for Childhood Asthma. N. Engl. J. Med..

[89] Bronfort G., Haas M., Evans R., Leininger B., Triano J. (2010). Effectiveness of manual therapies: The UK evidence report. Chiropr. Osteopat..

[90] Hondras M.A., Linde K., Jones A.P. (2002). Manual therapy for asthma. Cochrane Database Syst Rev..

[91] Kaminskyj A., Frazier M., Johnstone K., Gleberzon B.J. (2010). Chiropr.actic care for patients with asthma: A systematic review of the literature. J. Can. Chiropr. Assoc..

[92] Ernst E. (2009). Spinal manipulation for asthma: A systematic review of randomised clinical trials. Respir. Med..

[93] Pearce B.D., Biron C.A., Miller A.H. (2001). Neuroendocrine-immune interactions during viral infections. Advances in Virus Research.

[94] Thayer J.F. (2009). Vagal tone and the inflammatory reflex. Cleve Clin. J. Med..

[95] Pavlov V.A., Wang H., Czura C.J., Friedman S.G., Tracey K.J. (2003). The cholinergic anti-inflammatory pathway: A missing link in neuroimmunomodulation. Mol. Med..

[96] Felten S.Y., Madden K.S., Bellinger D.L., Kruszewska B., Moynihan J.A., Felten D.L., Goldstein D.S., Eisenhofer G., McCarty R. (1997). The Role of the Sympathetic Nervous System in the Modulation of Immune Responses. Advances in Pharmacology.

[97] Kohm A.P., Sanders V.M. (2000). Norepinephrine: A messenger from the brain to the immune system. Immunol. Today.

[98] Kiecolt-Glaser J.K., Gouin J.-P., Hantsoo L. (2010). Close relationships, inflammation, and health. Neurosci. Biobehav. Rev..

[99] Pavlov V.A., Tracey K.J. (2012). The vagus nerve and the inflammatory reflex—linking immunity and metabolism. Nat. Rev. Endocrinol..

[100] Felitti V.J., Anda R.F., Nordenberg D., Williamson D.F., Spitz A.M., Edwards V., Koss M.P., Marks J.S. (2019). Relationship of childhood abuse and household dysfunction to many of the leading causes of death in adults: The Adverse Childhood Experiences (ACE) Study. Am. J. Prev. Med..

[101] Dube S.R., Fairweather D., Pearson W.S., Felitti V.J., Anda R.F., Croft J.B. (2009). Cumulative childhood stress and autoimmune diseases in adults. Psychosom. Med..

[102] Naudé P.J., Roest A.M., Stein D.J., de Jonge P., Doornbos B. (2018). Anxiety disorders and CRP in a population cohort study with 54,326 participants: The LifeLines study. World J. Biol. Psychiatry.

[103] White J., Kivimäki M., Jokela M., Batty G.D. (2017). Association of inflammation with specific symptoms of depression in a general population of older people: The English Longitudinal Study of Ageing. Brain Behav. Immun..

[104] Melhem N.M., Munroe S., Marsland A., Gray K., Brent D., Porta G., Douaihy A., Laudenslager M.L., DePietro F., Diler R. (2017). Blunted HPA axis activity prior to suicide attempt and increased inflammation in attempters. Psychoneuroendocrinology.

[105] Leonard B.E. (2018). Inflammation and depression: A causal or coincidental link to the pathophysiology?. Acta Neuropsychiatr..

[106] Köhler-Forsberg O., Buttenschøn H.N., Tansey K.E., Maier W., Hauser J., Dernovsek M.Z., Henigsberg N., Souery D., Farmer A., Rietschel M. (2017). Association between C-reactive protein (CRP) with depression symptom severity and specific depressive symptoms in major depression. Brain Behav. Immun..

[107] Tayefi M., Shafiee M., Kazemi-Bajestani S.M.R., Esmaeili H., Darroudi S., Khakpouri S., Mohammadi M., Ghaneifar Z., Azarpajouh M.R., Moohebati M. (2017). Depression and anxiety both associate with serum level of hs-CRP: A gender-stratified analysis in a population-based study. Psychoneuroendocrinology.

[108] Tabatabaeizadeh S.-A., Abdizadeh M.F., Meshkat Z., Khodashenas E., Darroudi S., Fazeli M., Ferns G.A., Avan A., Ghayour-Mobarhan M. (2018). There is an association between serum high-sensitivity C-reactive protein (hs-CRP) concentrations and depression score in adolescent girls. Psychoneuroendocrinology.

[109] Smagula S.F., Ancoli-Israel S., Barrett-Connor E., Lane N.E., Redline S., Stone K.L., Cauley J.A., Group O.F.i.M.R. (2014). Inflammation, sleep disturbances, and depressed mood among community-dwelling older men. J. Psychosom. Res..

[110] Serrats J., Grigoleit J.-S., Alvarez-Salas E., Sawchenko P.E. (2017). Pro-inflammatory immune-to-brain signaling is involved in neuroendocrine responses to acute emotional stress. Brain Behav. Immun..

[111] Serafini G., Pompili M., Seretti M.E., Stefani H., Palermo M., Coryell W., Girardi P. (2013). The role of inflammatory cytokines in suicidal behavior: A systematic review. Eur. Neuropsychopharmacol..

[112] O’Donovan A., Rush G., Hoatam G., Hughes B.M., McCrohan A., Kelleher C., O’Farrelly C., Malone K.M. (2013). Suicidal ideation is associated with elevated inflammation in patients with major depressive disorder. Depress. Anxiety.

[113] Rotenberg S., McGrath J.J. (2016). Inter-relation between autonomic and HPA axis activity in children and adolescents. Biol. Psychol..

[114] Picchiottino M., Honoré M., Leboeuf-Yde C., Gagey O., Cottin F., Hallman D.M. (2020). The effect of a single spinal manipulation on cardiovascular autonomic activity and the relationship to pressure pain threshold: A randomized, cross-over, sham-controlled trial. Chiropr. Man. Ther..

[115] Picchiottino M., Leboeuf-Yde C., Gagey O., Hallman D.M. (2019). The acute effects of joint manipulative techniques on markers of autonomic nervous system activity: A systematic review and meta-analysis of randomized sham-controlled trials. Chiropr. Man. Ther..

[116] Kipnis J. (2018). The Seventh Sense. Sci. Am..

[117] Elenkov I.J., Wilder R.L., Chrousos G.P., Vizi E.S. (2000). The sympathetic nerve—an integrative interface between two supersystems: The brain and the immune system. Pharmacol. Rev..

[118] Baarbé J., Yielder P., Haavik H., Holmes M., Debison-Larabie C., Murphy B. Enhanced cerebellar disinhibition when cervical manipulation precedes motor learning in patients with subclinical neck pain. Proceedings of the WFC 13th Biennial Congress Proceedings.

[119] Baarbé J.K., Yielder P., Haavik H., Holmes M.W., Murphy B.A. (2018). Subclinical recurrent neck pain and its treatment impacts motor training-induced plasticity of the cerebellum and motor cortex. PLoS ONE.

[120] Christiansen T.L., Niazi I.K., Holt K., Nedergaard R.W., Duehr J., Allen K., Marshall P., Türker K.S., Hartvigsen J., Haavik H. (2018). The effects of a single session of spinal manipulation on strength and cortical drive in athletes. Eur. J. Appl. Physiol..

[121] Daligadu J., Haavik H., Yielder P., Baarbe J., Murphy B. (2013). Alterations in cortical and cerebellar motor processingt in sub-clinical neck pain patients following spinal manipulation. J. Manip. Physiol. Ther..

[122] Haavik H., Murphy B. (2011). Subclinical neck pain and the effects of cervical manipulation on elbow joint position sense. J. Manip. Physiol. Ther..

[123] Haavik H., Murphy B. (2012). The role of spinal manipulation in addressing disordered sensorimotor integration and altered motor control. J. Electromyogr. Kinesiol..

[124] Haavik H., Niazi I., Jochumsen M., Sherwin D., Flavel S., Türker K. (2017). Impact of Spinal Manipulation on Cortical Drive to Upper and Lower Limb Muscles. Brain Sci..

[125] Haavik H., Niazi I.K., Holt K., Murphy B. (2017). Effects of 12 Weeks of Chiropr.actic Care on Central Integration of Dual Somatosensory Input in Chronic Pain Patients: A Preliminary Study. J. Manip. Physiol. Ther..

[126] Haavik H., Niazi I.K., Jochumsen M., Uginčius P., Sebik O., Yılmaz G., Navid M.S., Özyurt M.G., Türker K.S. (2018). Chiropr.actic spinal manipulation alters TMS induced I-wave excitability and shortens the cortical silent period. J. Electromyogr. Kinesiol..

[127] Haavik Taylor H., Murphy B. (2007). Transient modulation of intracortical inhibition following spinal manipulation. Chiropr. J. Aust..

[128] Haavik Taylor H., Murphy B. (2008). Altered sensorimotor integration with cervical spine manipulation. J. Manip. Physiol. Ther..

[129] Haavik Taylor H., Murphy B. (2010). Altered Central Integration of Dual Somatosensory Input Following Cervical Spine Manipulation. J. Manip. Physiol. Ther..

[130] Haavik Taylor H., Murphy B. (2010). The effects of spinal manipulation on central integration of dual somatosensory input observed following motor training: A crossover study. J. Manip. Physiol. Ther..

[131] Haavik Taylor H., Murphy B. (2007). Cervical spine manipulation alters sensorimotor integration: A somatosensory evoked potential study. Clin. Neurophysiol..

[132] Holt K., Haavik H., Murphy B., Chi Lun Lee A., Elley C. (2016). Effectiveness of Chiropr.actic Care to Improve Sensorimotor Function Associated with Falls Risk in Older People: A Randomized Controlled Trial. J. Manip. Physiol. Ther..

[133] Holt K., Niazi I.K., Nedergaard R.W., Duehr J., Amjad I., Shafique M., Anwar M.N., Ndetan H., Turker K.S., Haavik H. (2019). The effects of a single session of chiropractic care on strength, cortical drive, and spinal excitability in stroke patients. Sci. Rep..

[134] Lelic D., Niazi I.K., Holt K., Jochumsen M., Dremstrup K., Yielder P., Murphy B., Drewes A.M., Haavik H. (2016). Manipulation of Dysfunctional Spinal Joints Affects Sensorimotor Integration in the Prefrontal Cortex: A Brain Source Localization Study. Neural Plast..

[135] Niazi I.K., Türker K.S., Flavel S., Kinget M., Duehr J., Haavik H. (2015). Changes in H-reflex and V-waves following spinal manipulation. Exp. Brain Res..

[136] Haavik Taylor H., Holt K., Murphy B. (2010). Exploring the neuromodulatory effects of the vertebral subluxation and chiropractic care. Chiropr. J. Aust.

[137] Du Rose A., Breen A. (2016). Relationships between paraspinal muscle activity and lumbar inter-vertebral range of motion. Healthcare.

[138] Park K.-N., Kwon O.-Y., Kim S.-J., Kim S.-H. (2017). Asymmetry of neck motion and activation of the cervical paraspinal muscles during prone neck extension in subjects with unilateral posterior neck pain. J. Back Musculoskelet. Rehabil..

[139] Kent C. (1996). Models of vertebral subluxation: A review. J. Vertebr. Subluxation Res..

[140] Henderson C.N. (2012). The basis for spinal manipulation: Chiropr.actic perspective of indications and theory. J. Electromyogr. Kinesiol..

[141] Alcantara J., Alcantara J.D., Alcantara J., Anrig C., Plaugher G. (2013). Spinal Subluxation. Pediatric Chiropractic.

[142] Baarbe J.K., Holmes M.W., Murphy H.E., Haavik H., Murphy B.A. (2016). Influence of Subclinical Neck Pain on the Ability to Perform a Mental Rotation Task: A 4-Week Longitudinal Study With a Healthy Control Group Comparison. J. Manip. Physiol. Ther..

[143] Faw B. (2003). Pre-frontal executive committee for perception, working memory, attention, long-term memory, motor control, and thinking: A tutorial review. Conscious. Cogn..

[144] Moench K.M., Wellman C.L. (2015). Review article: Stress-induced alterations in prefrontal dendritic spines: Implications for post-traumatic stress disorder. Neurosci. Lett..

[145] Ahern G.L., Sollers J.J., Lane R.D., Labiner D.M., Herring A.M., Weinand M.E., Hutzler R., Thayer J.F. (2001). Heart rate and heart rate variability changes in the intracarotid sodium amobarbital test. Epilepsia.

[146] Berthoud H.-R., Neuhuber W.L. (2000). Functional and chemical anatomy of the afferent vagal system. Auton. Neurosci..

[147] Thayer J.F., Sternberg E.M. (2010). Neural aspects of immunomodulation: Focus on the vagus nerve. Brain Behav. Immun..

[148] Bankenahally R., Krovvidi H. (2016). Autonomic nervous system: Anatomy, physiology, and relevance in anaesthesia and critical care medicine. BJA Educ..

[149] Kul’chyns’kyi A.B., Kyjenko V.M., Zukow W., Popovych I.L. (2017). Causal neuro-immune relationships at patients with chronic pyelonephritis and cholecystitis. correlations between parameters EEG, HRV and white blood cell count. Open Med..

[150] Ohira H., Matsunaga M., Osumi T., Fukuyama S., Shinoda J., Yamada J., Gidron Y. (2013). Vagal nerve activity as a moderator of brain–immune relationships. J. Neuroimmunol..

[151] Thayer J.F., Åhs F., Fredrikson M., Sollers J.J., Wager T.D. (2012). A meta-analysis of heart rate variability and neuroimaging studies: Implications for heart rate variability as a marker of stress and health. Neurosci. Biobehav. Rev..

[152] McCraty R., Shaffer F. (2015). Heart Rate Variability: New Perspectives on Physiological Mechanisms, Assessment of Self-regulatory Capacity, and Health Risk. Glob. Adv. Health Med..

[153] Hänsel A., Von Känel R. (2008). The ventro-medial prefrontal cortex: A major link between the autonomic nervous system, regulation of emotion, and stress reactivity?. Biopsychosoc. Med..

[154] Diorio D., Viau V., Meaney M.J. (1993). The role of the medial prefrontal cortex (cingulate gyrus) in the regulation of hypothalamic-pituitary-adrenal responses to stress. J. Neurosci..

[155] Lorenz J., Minoshima S., Casey K. (2003). Keeping pain out of mind: The role of the dorsolateral prefrontal cortex in pain modulation. Brain.

[156] Apkarian A.V., Bushnell M.C., Treede R.D., Zubieta J.K. (2005). Human brain mechanisms of pain perception and regulation in health and disease. Eur. J. Pain: EJP.

[157] Apkarian A.V., Sosa Y., Sonty S., Levy R.M., Harden R.N., Parrish T.B., Gitelman D.R. (2004). Chronic Back Pain Is Associated with Decreased Prefrontal and Thalamic Gray Matter Density. J. Neurosci..

[158] Arnsten A.F.T., Raskind M.A., Taylor F.B., Connor D.F. (2015). blair. Neurobiol. Stress.

[159] Eden A.S., Schreiber J., Anwander A., Keuper K., Laeger I., Zwanzger P., Zwitserlood P., Kugel H., Dobel C. (2015). Emotion regulation and trait anxiety are predicted by the microstructure of fibers between amygdala and prefrontal cortex. J. Neurosci..

[160] Etkin A., Büchel C., Gross J.J. (2015). The neural bases of emotion regulation. Nat. Rev. Neurosci..

[161] Ghosal S., Hare B.D., Duman R.S. (2017). Prefrontal cortex GABAergic deficits and circuit dysfunction in the pathophysiology and treatment of chronic stress and depression. Curr. Opin. Behav. Sci..

[162] Johnston-Wilson N.L., Sims C.D., Hofmann J.P., Anderson L., Shore A.D., Torrey E.F., Yolken R.H. (2000). Disease-specific alterations in frontal cortex brain proteins in schizophrenia, bipolar disorder, and major depressive disorder. Mol. Psychiatry.

[163] Motzkin J.C., Philippi C.L., Wolf R.C., Baskaya M.K., Koenigs M. (2015). Ventromedial prefrontal cortex is critical for the regulation of amygdala activity in humans. Biol. Psychiatry.

[164] Kawli T., He F., Tan M.-W. (2010). It takes nerves to fight infections: Insights on neuro-immune interactions from C. elegans. Dis. Models Mech..

[165] Godinho-Silva C., Cardoso F., Veiga-Fernandes H. (2019). Neuro–immune cell units: A new paradigm in physiology. Annu. Rev. Immunol..

[166] Sanders V.M., Kohm A.P. (2002). Sympathetic nervous system interaction with the immune system. Int. Rev. Neurobiol..

[167] Sternberg E.M. (2006). Neural regulation of innate immunity: A coordinated nonspecific host response to pathogens. Nat. Rev. Immunol..

[168] Louveau A., Herz J., Alme M.N., Salvador A.F., Dong M.Q., Viar K.E., Herod S.G., Knopp J., Setliff J.C., Lupi A.L. (2018). CNS lymphatic drainage and neuroinflammation are regulated by meningeal lymphatic vasculature. Nat. Neurosci..

[169] Chiu I.M., Heesters B.A., Ghasemlou N., Von Hehn C.A., Zhao F., Tran J., Wainger B., Strominger A., Muralidharan S., Horswill A.R. (2013). Bacteria activate sensory neurons that modulate pain and inflammation. Nature.

[170] Schiller M., Ben-Shaanan T.L., Rolls A. (2020). Neuronal regulation of immunity: Why, how and where?. Nat. Rev. Immunol.

[171] Dantzer R. (2018). Neuroimmune interactions: From the brain to the immune system and vice versa. Physiol. Rev..

[172] Kamimura D., Ohki T., Arima Y., Ota M., Murakami M. (2019). Gateway reflex: Local neuroimmune interactions that regulate blood vessels. Neurochem. Int..

[173] Imeri L., Opp M.R. (2009). How (and why) the immune system makes us sleep. Nat. Rev. Neurosci..

[174] Krueger J.M. (2008). The role of cytokines in sleep regulation. Curr. Pharm. Des..

[175] Ahima R.S., Antwi D.A. (2008). Brain regulation of appetite and satiety. Endocrinol. Metab. Clin. N. Am..

[176] Buchanan J.B., Johnson R.W. (2007). Regulation of food intake by inflammatory cytokines in the brain. Neuroendocrinology.

[177] Marin I., Kipnis J. (2013). Learning and memory and the immune system. Learn. Mem..

[178] Filiano A.J., Xu Y., Tustison N.J., Marsh R.L., Baker W., Smirnov I., Overall C.C., Gadani S.P., Turner S.D., Weng Z. (2016). Unexpected role of interferon-γ in regulating neuronal connectivity and social behaviour. Nature.

[179] Bay-Richter C., Janelidze S., Hallberg L., Brundin L. (2011). Changes in behaviour and cytokine expression upon a peripheral immune challenge. Behav. Brain Res..

[180] Müller N., Riedel M., Gruber R., Ackenheil M., Schwarz M.J. (2000). The immune system and schizophrenia: An integrative view. Ann. N. Y. Acad. Sci..

[181] Heppner F.L., Ransohoff R.M., Becher B. (2015). Immune attack: The role of inflammation in Alzheimer disease. Nat. Rev. Neurosci..

[182] Enck P., Benedetti F., Schedlowski M. (2008). New insights into the placebo and nocebo responses. Neuron.

[183] Benedetti F. (2014). Placebo Effects.

[184] Wong C.H., Jenne C.N., Lee W.-Y., Léger C., Kubes P. (2011). Functional innervation of hepatic iNKT cells is immunosuppressive following stroke. Science.

[185] Miller H., Tilston J., Langhorne P., Stott D.J. (2007). Risk Factors for Chest Infection in Acute Stroke. Stroke.

[186] Moshel Y.A., Durkin H.G., Amassian V.E. (2005). Lateralized neocortical control of T lymphocyte export from the thymus: I. Increased export after left cortical stimulation in behaviorally active rats, mediated by sympathetic pathways in the upper spinal cord. J. Neuroimmunol..

[187] Neveu P., Barnéoud P., Vitiello S., Betancur C., Le Moal M. (1988). Brain modulation of the immune system: Association between lymphocyte responsiveness and paw preference in mice. Brain Res..

[188] Betancur C., Neveu P.J., Vitiello S., Le Moal M. (1991). Natural killer cell activity is associated with brain asymmetry in male mice. Brain Behav. Immun..

[189] Neveu P. (1992). Asymmetrical brain modulation of the immune response. Brain Res. Rev..

[190] Tarkowski E., Ekelund P., Tarkowski A. (1991). Enhancement of antigen-specific T-cell reactivity on the affected side in stroke patients. J. Neuroimmunol..

[191] Wittling W., Block A., Schweiger E., Genzel S. (1998). Hemisphere asymmetry in sympathetic control of the human myocardium. Brain Cogn..

[192] Meyer S., Strittmatter M., Fischer C., Georg T., Schmitz B. (2004). Lateralization in autononic dysfunction in ischemic stroke involving the insular cortex. Neuroreport.

[193] Guo C.C., Sturm V.E., Zhou J., Gennatas E.D., Trujillo A.J., Hua A.Y., Crawford R., Stables L., Kramer J.H., Rankin K. (2016). Dominant hemisphere lateralization of cortical parasympathetic control as revealed by frontotemporal dementia. Proc. Natl. Acad. Sci. USA.

[194] Wittling W., Block A., Genzel S., Schweiger E. (1998). Hemisphere asymmetry in parasympathetic control of the heart. Neuropsychologia.

[195] Aich P., Potter A.A., Griebel P.J. (2009). Modern approaches to understanding stress and disease susceptibility: A review with special emphasis on respiratory disease. Int. J. Gen. Med..

[196] Dorshkind K., Horseman N.D. (2001). Anterior pituitary hormones, stress, and immune system homeostasis. BioEssays.

[197] Leonard B.E. (2006). HPA and Immune Axes in Stress: Involvement of the Serotonergic System. Neuroimmunomodulation.

[198] Meisel C., Schwab J.M., Prass K., Meisel A., Dirnagl U. (2005). Central nervous system injury-induced immune deficiency syndrome. Nat. Rev. Neurosci..

[199] Piedimonte G. (2003). Contribution of neuroimmune mechanisms to airway inflammation and remodeling during and after respiratory syncytial virus infection. Pediatr. Infect. Dis. J..

[200] Shimada A., Yokota T. (2020). Physiological and pathological brain-immune system interactions. Clin. Exp. Neuroimmunol..

[201] Silverman M.N., Pearce B.D., Biron C.A., Miller A.H. (2005). Immune modulation of the hypothalamic-pituitary-adrenal (HPA) axis during viral infection. Viral Immunol..

[202] Sharma H.S., Winkler T., Stålberg E., Mohanty S., Westman J. (2000). p-Chlorophenylalanine, an inhibitor of serotonin synthesis reduces blood-brain barrier permeability, cerebral blood flow, edema formation and cell injury following trauma to the rat brain. Brain Edema XI.

[203] Korin B., Avraham S., Azulay-Debby H., Farfara D., Hakim F., Rolls A. (2020). Short-term sleep deprivation in mice induces B cell migration to the brain compartment. Sleep.

[204] Kertser A., Baruch K., Deczkowska A., Weiner A., Croese T., Kenigsbuch M., Cooper I., Tsoory M., Ben-Hamo S., Amit I. (2019). Corticosteroid signaling at the brain-immune interface impedes coping with severe psychological stress. Sci. Adv..

[205] Liu Y.U., Ying Y., Li Y., Eyo U.B., Chen T., Zheng J., Umpierre A.D., Zhu J., Bosco D.B., Dong H. (2019). Neuronal network activity controls microglial process surveillance in awake mice via norepinephrine signaling. Nat. Neurosci..

[206] Stowell R.D., Sipe G.O., Dawes R.P., Batchelor H.N., Lordy K.A., Whitelaw B.S., Stoessel M.B., Bidlack J.M., Brown E., Sur M. (2019). Noradrenergic signaling in the wakeful state inhibits microglial surveillance and synaptic plasticity in the mouse visual cortex. Nat. Neurosci..

[207] Van Wagoner N.J., Benveniste E.N. (1999). Interleukin-6 expression and regulation in astrocytes. J. Neuroimmunol..

[208] Cannella B., Raine C.S. (2004). Multiple sclerosis: Cytokine receptors on oligodendrocytes predict innate regulation. Ann. Neurol..

[209] Vezzani A., Viviani B. (2015). Neuromodulatory properties of inflammatory cytokines and their impact on neuronal excitability. Neuropharmacology.

[210] Cámara-Lemarroy C.R., Cordero-Perez P., Ibarra-Hernandez J.M., Muñoz-Espinosa L.E., Fernandez-Garza N.E. (2015). Gemfibrozil attenuates the inflammatory response and protects rats from abdominal sepsis. Exp. Ther. Med..

[211] Budd A., Alleva L., Alsharifi M., Koskinen A., Smythe V., Müllbacher A., Wood J., Clark I. (2007). Increased survival after gemfibrozil treatment of severe mouse influenza. Antimicrob. Agents Chemother..

[212] Demas G.E. (2004). The energetics of immunity: A neuroendocrine link between energy balance and immune function. Horm. Behav..

[213] Derting T.L., Compton S. (2003). Immune response, not immune maintenance, is energetically costly in wild white-footed mice (Peromyscus leucopus). Physiol. Biochem. Zool..

[214] Muehlenbein M.P., Hirschtick J.L., Bonner J.Z., Swartz A.M. (2010). Toward quantifying the usage costs of human immunity: Altered metabolic rates and hormone levels during acute immune activation in men. Am. J. Hum. Biol. J. Hum. Biol. Assoc..

[215] Gillette M.U., Tischkau S.A. (1999). Suprachiasmatic nucleus: The brain’s circadian clock. Recent Prog. Horm. Res..

[216] Scheiermann C., Kunisaki Y., Frenette P.S. (2013). Circadian control of the immune system. Nat. Rev. Immunol..

[217] Druzd D., Matveeva O., Ince L., Harrison U., He W., Schmal C., Herzel H., Tsang A.H., Kawakami N., Leliavski A. (2017). Lymphocyte circadian clocks control lymph node trafficking and adaptive immune responses. Immunity.

[218] Allen G.V., Saper C.B., Hurley K.M., Cechetto D.F. (1991). Organization of visceral and limbic connections in the insular cortex of the rat. J. Comp. Neurol..

[219] Craig A.D. (2003). Interoception: The sense of the physiological condition of the body. Curr. Opin. Neurobiol..

[220] Pacheco-López G., Niemi M.-B., Kou W., Härting M., Fandrey J., Schedlowski M. (2005). Neural substrates for behaviorally conditioned immunosuppression in the rat. J. Neurosci..

[221] Ader R., Cohen N. (1975). Behaviorally conditioned immunosuppression. Psychosom. Med..

[222] Hyman S.E., Malenka R.C., Nestler E.J. (2006). Neural mechanisms of addiction: The role of reward-related learning and memory. Annu. Rev. Neurosci..

[223] Russo S.J., Nestler E.J. (2013). The brain reward circuitry in mood disorders. Nat. Rev. Neurosci..

[224] Tsai H.-C., Zhang F., Adamantidis A., Stuber G.D., Bonci A., De Lecea L., Deisseroth K. (2009). Phasic firing in dopaminergic neurons is sufficient for behavioral conditioning. Science.

[225] Ben-Shaanan T.L., Azulay-Debby H., Dubovik T., Starosvetsky E., Korin B., Schiller M., Green N.L., Admon Y., Hakim F., Shen-Orr S.S. (2016). Activation of the reward system boosts innate and adaptive immunity. Nat. Med..

[226] Pinho-Ribeiro F.A., Verri W.A., Chiu I.M. (2017). Nociceptor sensory neuron–immune interactions in pain and inflammation. Trends Immunol..

[227] Hoffman K.E., Maslonek K.A., Dykstra L.A., Lysle D.T. (1995). Effects of central administration of morphine on immune status in Lewis and Wistar rats. The Brain Immune Axis and Substance Abuse.

[228] Gomez-Flores R., Weber R.J. (1999). Inhibition of interleukin-2 production and downregulation of IL-2 and transferrin receptors on rat splenic lymphocytes following PAG morphine administration: A role in natural killer and T cell suppression. J. Interferon Cytokine Res..

[229] Gomez-Flores R., Suo J., Weber R.J. (1999). Suppression of splenic macrophage functions following acute morphine action in the rat mesencephalon periaqueductal gray. Brain Behav. Immun..

[230] Demetrikopoulos M.K., Siegel A., Schleifer S.J., Obedi J., Keller S.E. (1994). Electrical stimulation of the dorsal midbrain periaqueductal gray suppresses peripheral blood natural killer cell activity. Brain Behav. Immun..

[231] Bushnell M.C., Čeko M., Low L.A. (2013). Cognitive and emotional control of pain and its disruption in chronic pain. Nat. Rev. Neurosci..

[232] Dhabhar F.S., Malarkey W.B., Neri E., McEwen B.S. (2012). Stress-induced redistribution of immune cells—From barracks to boulevards to battlefields: A tale of three hormones–Curt Richter Award Winner. Psychoneuroendocrinology.

[233] Schedlowski M., Jacobs R., Stratmann G., Richter S., Hädicke A., Tewes U., Wagner T.O., Schmidt R.E. (1993). Changes of natural killer cells during acute psychological stress. J. Clin. Immunol..

[234] Nance D.M., Sanders V.M. (2007). Autonomic innervation and regulation of the immune system (1987–2007). Brain Behav. Immun..

[235] Elmquist J.K., Scammell T.E., Jacobson C.D., Saper C.B. (1996). Distribution of Fos-like immunoreactivity in the rat brain following intravenous lipopolysaccharide administration. J. Comp. Neurol..

[236] Soto-Tinoco E., Guerrero-Vargas N.N., Buijs R.M. (2016). Interaction between the hypothalamus and the immune system. Exp. Physiol..

[237] Hefco V., Olariu A., Hefco A., Nabeshima T. (2004). The modulator role of the hypothalamic paraventricular nucleus on immune responsiveness. Brain Behav. Immun..

[238] Sakic B., Vlajkovic S. (1990). Self-stimulation behavior: Consequences upon immunity?. Brain Behav. Immun..

[239] Wrona D., Jurkowski M., Luszawska D., Tokarski J., Trojniar W. (2003). The effects of lateral hypothalamic lesions on peripheral blood natural killer cell cytotoxicity in rats hyper-and hyporesponsive to novelty. Brain Behav. Immun..

[240] Churchland P.S., Winkielman P. (2012). Modulating social behavior with oxytocin: How does it work? What does it mean?. Horm. Behav..

[241] Neumann I.D. (2008). Brain oxytocin: A key regulator of emotional and social behaviours in both females and males. J. Neuroendocrinol..

[242] Boone M., Deen P.M. (2008). Physiology and pathophysiology of the vasopressin-regulated renal water reabsorption. Pflügers Arch. Eur. J. Physiol..

[243] Butterworth M., McClellan B., Aklansmith M. (1967). Influence of sex on immunoglobulin levels. Nature.

[244] Von Haam E., Rosenfeld I. (1942). The effect of estrone on antibody-production. J. Immunol..

[245] Mangalam A.K., Taneja V., David C.S. (2013). HLA class II molecules influence susceptibility versus protection in inflammatory diseases by determining the cytokine profile. J. Immunol..

[246] Ngo S.T., Steyn F.J., McCombe P.A. (2014). Gender differences in autoimmune disease. Front. Neuroendocrinol..

[247] Barreiro Arcos M.a.L., Gorelik G., Klecha A., Genaro A.M., Cremaschi G.A. (2006). Thyroid hormones increase inducible nitric oxide synthase gene expression downstream from PKC-ζ in murine tumor T lymphocytes. Am. J. Physiol. Cell Physiol..

[248] Klecha A.J., Genaro A.M., Gorelik G., Arcos M.L.B., Silberman D.M., Schuman M., Garcia S.I., Pirola C., Cremaschi G.A. (2006). Integrative study of hypothalamus–pituitary–thyroid–immune system interaction: Thyroid hormone-mediated modulation of lymphocyte activity through the protein kinase C signaling pathway. J. Endocrinol..

[249] Tan T.L., Rajeswaran H., Haddad S., Shahi A., Parvizi J. (2016). Increased risk of periprosthetic joint infections in patients with hypothyroidism undergoing total joint arthroplasty. J. Arthroplast..

[250] Nóbrega M.M., Auge A.P.F., de Toledo L.G.M., da Silva Carramão S., Frade A.B., Salles M.J.C. (2015). Bacteriuria and urinary tract infection after female urodynamic studies: Risk factors and microbiological analysis. Am. J. Infect. Control..

[251] Baker E., Lui F. (2019). Neuroanatomy, Vagal Nerve Nuclei (Nucleus Vagus).

[252] Guyenet P.G. (2006). The sympathetic control of blood pressure. Nat. Rev. Neurosci..

[253] Samuels E., Szabadi E. (2008). Functional neuroanatomy of the noradrenergic locus coeruleus: Its roles in the regulation of arousal and autonomic function part II: Physiological and pharmacological manipulations and pathological alterations of locus coeruleus activity in humans. Curr. Neuropharmacol..

[254] Jansen A.S., Van Nguyen X., Karpitskiy V., Mettenleiter T.C., Loewy A.D. (1995). Central command neurons of the sympathetic nervous system: Basis of the fight-or-flight response. Science.

[255] Kanbar R., Depuy S.D., West G.H., Stornetta R.L., Guyenet P.G. (2011). Regulation of visceral sympathetic tone by A5 noradrenergic neurons in rodents. J. Physiol..

[256] Nakamura K., Matsumura K., Kaneko T., Kobayashi S., Katoh H., Negishi M. (2002). The rostral raphe pallidus nucleus mediates pyrogenic transmission from the preoptic area. J. Neurosci..

[257] Morrison S.F., Sved A.F., Passerin A.M. (1999). GABA-mediated inhibition of raphe pallidus neurons regulates sympathetic outflow to brown adipose tissue. Am. J. Physiol. Regul. Integr. Comp. Physiol..

[258] Navarro X., Verdu E., Wendelschafer-Crabb G., Kennedy W. (1995). Innervation of cutaneous structures in the mouse hind paw: A confocal microscopy immunohistochemical study. J. Neurosci. Res..

[259] Ishida-Yamamoto A., Senba E., Tohyama M. (1989). Distribution and fine structure of calcitonin gene-related peptide-like immunoreactive nerve fibers in the rat skin. Brain Res..

[260] Arnsten A.F. (2009). Stress signalling pathways that impair prefrontal cortex structure and function. Nat. Rev. Neurosci..

[261] Amat J., Baratta M.V., Paul E., Bland S.T., Watkins L.R., Maier S.F. (2005). Medial prefrontal cortex determines how stressor controllability affects behavior and dorsal raphe nucleus. Nat. Neurosci..

[262] Arco A.D., Mora F. (2009). Neurotransmitters and prefrontal cortex–limbic system interactions: Implications for plasticity and psychiatric disorders. J. Neural Transm..

[263] Elstad J.I., Vabø M. (2008). Job stress, sickness absence and sickness presenteeism in Nordic elderly care. Scand. J. Public Health.

[264] Johnson J.D., O’Connor K.A., Hansen M.K., Watkins L.R., Maier S.F. (2003). Effects of prior stress on LPS-induced cytokine and sickness responses. Am. J. Physiol. Regul. Integr. Comp. Physiol..

[265] Wulsin L.R., Vaillant G.E., Wells V.E. (1999). A systematic review of the mortality of depression. Psychosom. Med..

[266] Eyre H., Baune B.T. (2012). Neuroplastic changes in depression: A role for the immune system. Psychoneuroendocrinology.

[267] Pittenger C., Duman R.S. (2008). Stress, depression, and neuroplasticity: A convergence of mechanisms. Neuropsychopharmacology.

[268] Cohen S., Tyrrell D.A.J., Smith A.P. (1991). Psychological Stress and Susceptibility to the Common Cold. N. Engl. J. Med..

[269] Danese A., Caspi A., Williams B., Ambler A., Sugden K., Mika J., Werts H., Freeman J., Pariante C., Moffitt T. (2011). Biological embedding of stress through inflammation processes in childhood. Mol. Psychiatry.

[270] Danese A., Moffitt T.E., Harrington H., Milne B.J., Polanczyk G., Pariante C.M., Poulton R., Caspi A. (2009). Adverse childhood experiences and adult risk factors for age-related disease: Depression, inflammation, and clustering of metabolic risk markers. Arch. Pediatr. Adolesc. Med..

[271] Danese A., Pariante C.M., Caspi A., Taylor A., Poulton R. (2007). Childhood maltreatment predicts adult inflammation in a life-course study. Proc. Natl. Acad. Sci. USA.

[272] Ershler W.B., Keller E.T. (2000). Age-associated increased interleukin-6 gene expression, late-life diseases, and frailty. Annu. Rev. Med..

[273] Viswanathan K., Dhabhar F.S. (2005). Stress-induced enhancement of leukocyte trafficking into sites of surgery or immune activation. Proc. Natl. Acad. Sci. USA.

[274] Dhabhar F.S., McEwen B.S. (1997). Acute stress enhances while chronic stress suppresses cell-mediated immunityin vivo: A potential role for leukocyte trafficking. Brain Behav. Immun..

[275] Dhabhar F.S. (2000). Acute stress enhances while chronic stress suppresses skin immunity: The role of stress hormones and leukocyte trafficking. Ann. N. Y. Acad. Sci..

[276] Kiecolt-Glaser J.K., Glaser R., Gravenstein S., Malarkey W.B., Sheridan J. (1996). Chronic stress alters the immune response to influenza virus vaccine in older adults. Proc. Natl. Acad. Sci. USA.

[277] Naliboff B.D., Benton D., Solomon G.F., Morley J.E., Fahey J.L., Bloom E.T., Makinodan T., Gilmore S.L. (1991). Immunological changes in young and old adults during brief laboratory stress. Psychosom. Med..

[278] Cole S.W. (2014). Human social genomics. PLoS Genet..

[279] Baral P., Umans B.D., Li L., Wallrapp A., Bist M., Kirschbaum T., Wei Y., Zhou Y., Kuchroo V.K., Burkett P.R. (2018). Nociceptor sensory neurons suppress neutrophil and γδ T cell responses in bacterial lung infections and lethal pneumonia. Nat. Med..

[280] Kihara N., De la Fuente S., Fujino K., Takahashi T., Pappas T., Mantyh C. (2003). Vanilloid receptor-1 containing primary sensory neurones mediate dextran sulphate sodium induced colitis in rats. Gut.

[281] McEwen B.S., Stellar E. (1993). Stress and the individual: Mechanisms leading to disease. Arch. Intern. Med..

[282] Dirnagl U., Klehmet J., Braun J.S., Harms H., Meisel C., Ziemssen T., Prass K., Meisel A. (2007). Stroke-induced immunodepression: Experimental evidence and clinical relevance. Stroke.

[283] Jones G., Cale A. (1997). Goal difficulty, anxiety and performance. Ergonomics.

[284] Van Galen G.P., van Huygevoort M. (2000). Error, stress and the role of neuromotor noise in space oriented behaviour. Biol. Psychol..

[285] Weinberg R.S., Hunt V.V. (1976). The interrelationships between anxiety, motor performance and electromyography. J. Mot. Behav..

[286] Marras W.S., Davis K.G., Heaney C.A., Maronitis A.B., Allread W.G. (2000). The influence of psychosocial stress, gender, and personality on mechanical loading of the lumbar spine. Spine.

[287] Ekberg K., Eklund J., Tuvesson M.-A., Örtengren R., Odenrick P., Ericson M. (1995). Psychological stress and muscle activity during data entry at visual display units. Work Stress.

[288] Butler D., Moseley G.L. (2003). Explain Pain.

[289] Shahidi B., Haight A., Maluf K. (2013). Differential effects of mental concentration and acute psychosocial stress on cervical muscle activity and posture. J. Electromyogr. Kinesiol..

[290] Marker R.J., Campeau S., Maluf K.S. (2017). Psychosocial stress alters the strength of reticulospinal input to the human upper trapezius. J. Neurophysiol..

[291] Hellström F., Roatta S., Thunberg J., Passatore M., Djupsjöbacka M. (2005). Responses of muscle spindles in feline dorsal neck muscles to electrical stimulation of the cervical sympathetic nerve. Exp. Brain Res..

[292] Passatore M., Roatta S. (2006). Influence of sympathetic nervous system on sensorimotor function: Whiplash associated disorders (WAD) as a model. Eur. J. Appl. Physiol..

[293] Flor H., Birbaumer N., Schugens M.M., Lutzenberger W. (1992). Symptom-Specific Psychophysiological Responses in Chronic Pain Patients. Psychophysiology.

[294] Vlaeyen J.W., Seelen H.A., Peters M., de Jong P., Aretz E., Beisiegel E., Weber W.E. (1999). Fear of movement/(re) injury and muscular reactivity in chronic low back pain patients: An experimental investigation. Pain.

[295] Hodges P.W., Moseley G.L. (2003). Pain and motor control of the lumbopelvic region: Effect and possible mechanisms. J. Electromyogr. Kinesiol..

[296] Biedermann H., Shanks G., Forrest W., Inglis J. (1991). Power spectrum analyses of electromyographic activity. Discriminators in the differential assessment of patients with chronic low-back pain. Spine.

[297] Watson P.J., Booker C.K., Main C.J. (1997). Evidence for the role of psychological factors in abnormal paraspinal activity in patients with chronic low back pain. J. Musculoskelet. Pain.

[298] Nederhand M.J., Hermens H.J., IJzerman M.J., Groothuis K.G., Turk D.C. (2006). The effect of fear of movement on muscle activation in posttraumatic neck pain disability. Clin. J. Pain.

[299] Karayannis N.V., Smeets R.J., van den Hoorn W., Hodges P.W. (2013). Fear of movement is related to trunk stiffness in low back pain. PLoS ONE.

[300] The Rubicon Group (2017). Definition and Position Statement on the Chiropractic Subluxation. https://www.therubicongroup.org/policies/.

[301] Kawchuk G., Goertz C., Axén I., Descarreaux M., French S., Haas M., Hartvigsen J., Kolberg C., Maiers M. (2020). The Effect of Spinal Adjustment/Manipulation on Immunity and the Immune System: A Rapid Review of Relevant Literature. https://www.wfc.org/website/images/wfc/Latest_News_and_Features/Spinal_Manipulation_Immunity_Review_2020_03_19.pdf.

