# Supplementary material for: The Potential Mechanisms of High-Velocity, Low-Amplitude, Controlled Vertebral Thrusts on Neuroimmune Function: A Narrative Review
Source: Medicina (Kaunas). 2021 May 27;57(6):536. doi: 10.3390/medicina57060536 (PMC8226758; doi:10.3390/medicina57060536)
Supplement: Supplementary file 1 [file medicina-57-00536-s001.zip › medicina-1236518-supplementary.pdf]

Supplementary File

Supplementary S1

Topic: The potential mechanisms of high-velocity, low-amplitude, controlled vertebral thrusts on neuroimmune function: A narrative review

Key concept:

- Chiropractic
- Immune system

Key terms

chiropractic or "spinal manipulation" or "vertebral manipulation" or "spinal manipulative therapy" or thrust or osteopath* or "manual therapy" or "spinal manipulative" or "high-velocity low-amplitude thrust" or hvla or "chiropractic spinal adjustments" or "chiropractic spinal adjustment" or "chiropractic spinal" or "spinal adjustment" or "chiropractic adjustment" or "chiropractic manipulation" or "spinal manipulation" or "osteopathic manipulation" or "manipulative treatment" or "HVLA-SM"

AND

"biological markers" or "pain markers" or "biochemical markers" or "stress biomarker" or endocrine* or "sympathetic nervous system" or hormone or cortisol or oxytocin or endorphins or catecholamine or neuropeptide or ACTH or "white blood cells" or "CD4" or "T-lymphocyte" or "lymphocyte" or "immune system" or "immune function" or immune* or "immune response"

Supplementary S2

Table 1 The effect of high-velocity-low-amplitude controlled vertebral thrusts on immune and endocrine markers. TNF: tumour necrosis factor, IL: interleukin, CRP: C-reactive protein. Shaded rows show publications with no reported changes after high-velocity, low-amplitude vertebral thrusts.

Reference	Participants (n=sample size)	Outcome measures	Intervention	Results
Christian et al 1988	Healthy (n = 20) and symptomatic cervical or thoracic pain (n= 20)	β-endorphin, cortisol and adrenocorticotropic hormone.	A single session of high - velocity, low-amplitude thrusts was given by a chiropractor at the involved vertebral segment(s) throughout the spine after soft tissue tension was reached. For exact levels of spinal fixations, refer to table 1, 2 and 3 in the paper (Christian et al 1988). Occurrence of an audible release was recorded.	No difference in sham and experimental group for concentration of β-endorphin, cortisol and adrenocorticotropic hormone but cortisol levels dropped over time (recorded 5 and 30 minutes postintervention) in all groups.
Luisetto et al 1982	Women with cervical arthrosis and Barres's syndrome (n=11)	Plasma levels of β-endorphin and calcitonin	10 consecutive sessions of "manipulative treatment" was delivered by a chiropractor. No details are given about what the "manipulative treatment" encompassed. The exact site of manipulation is not given.	There was no significant change in plasma levels of β-endorphin following the manipulative treatment. A significant within group decrease in plasma calcitonin level was found after the intervention when dosed with antiserum A, while the levels did not change when dosed with antiserum B.
Brennan et al 1991	Healthy (n= 99)	Zymosan-stimulated chemiluminescenc	Experimental group (n=42). A single high-velocity, low -amplitude	The CL responses of both PMN and monocytes from subjects who received thoracic high-velocity, low -amplitude

		e (CL) responses of polymorphonuclear neutrophils (PMN) and monocytes<br><br>Plasma levels of substance P from two subsets of participants	thrust that produced an auditory release or palpable joint movement was delivered by a chiropractor over segments exhibiting the least flexibility (T2-T6) identified by motion palpation of intersegmental motion and soft-tissue palpation. Mean ± Standard deviation of the amplitude of the thrust was 676±55 Newtons. Rise times from preload to maximum force level was 136±20mSec.<br><br>Sham (n=38): A single low-velocity light-force thrust to the selected segment (No detail on the selected segment is given by authors). Mean ± standard deviation of the amplitude of the thrust was 241±26 Newtons. Rise times from preload to	thrust were significantly higher 15 minutes after than before treatment, and significantly higher than the response in sham or soft-tissue treated subjects. Plasma levels of SP before and after treatment in sham treated subjects did not differ significantly; however, elevated plasma SP was observed in subjects after thoracic high-velocity, low - amplitude thrust.

			maximum force level was 135±6mSec.<br><br>"Soft-tissue manipulation (n=19): Five thrusts to either the right or left gluteal area"	
Brennan et al 1994	Chronic low back pain (n=201).<br>In all, 148 cases were analyzed for B cells, 146 for T-Helper (TH), T-Suppressor (TS) and Natural Killer (NK) cells and 138 for cells that carried both the NK and TS marker.	Absolute numbers and percentages of B-lymphocytes, T-lymphocytes, T_H, TS and NK lymphocytes collected at the initial visit, on the final treatment session (12th visit) and follow-up after two weeks of no treatment.	Experimental group: Eleven sessions (over two weeks) of high-force, high-velocity, low-amplitude thrust procedure was delivered by a chiropractor to all levels of the spine between T12 and S1 and including sacroiliac joints that were clinically relevant for the patient.<br><br>Sham: Eleven sessions (over two weeks) of low-force, high-velocity, low-amplitude procedure was delivered by a chiropractor to a single spinal level of the lumbar spine. Reason why it was delivered to	"All subpopulation baseline values were within reported reference ranges for normal adult populations. However, the percentage of NK cells (9.1%) was below the published minimum critical value. The cell types which increased and for which the interaction tests were at or near statistical significance were: T_H cells ($p = .0208$), total T cell percent ($p = .0928$) but absolute total T cells decreased at 12th visit ($p = .0908$). Interaction tests for differences in either percent or absolute counts of B cells, TS cells, or NK cells did not change and were not statistically significant."

			the lumbar spine is not given.<br><br>A series of educational lectures on lower back pain.	
Selano et al 1994	HIV positive patients (n = 10)	CD4 Count	Single group study. The Grostic method of analysis and adjusting the upper cervical spine was used over a six month period.<br>Before the first intervention session, each participant underwent a physical examination involving visceral, orthopedic, chiropractic, range of motion and historical findings.	A 48% increase in CD4 cells was reported after the six-month duration of the study.
Tuchin 1998	Corporate Employees (n = 9)	Salivary Cortisol	Four spinal thrusts over a period of two weeks were performed by the chiropractor at vertebral levels determined to be restricted in motion as determined by orthopaedic and physical tests. No details given on	No significant change in concentration of salivary cortisol before and after the intervention.

			the exact spinal segments adjusted.	
Whelan 2002	Healthy (n= 30)	Salivary cortisol levels	A single session of high-velocity, low-amplitude thrusts was delivered by a chiropractor. Particularly, supine, coupled lateral flexion-rotational thrust for the upper cervical region was delivered unilaterally to the right side of the spine. The reason why this segment was chosen for the thrust is not given.	No effect on salivary cortisol levels in asymptomatic subjects.
Davison & Parkin-Smith, 2003	People with clinical evidence of upper cervical spine dysfunction without pain (n=10)	Lymphocyte activation	Intervention group (n=7): A single session of high-velocity, low-amplitude thrust was delivered by a chiropractor at the level (s) of joint (upper cervical spine) dysfunction identified by using the criteria stated by Bergman et al, (1993). A rotary and lateral break technique in the direction of joint restriction was used.	The results suggest that the lymphocytic response increased post sham session and reduced post high-velocity, low-amplitude thrust, but this could be a chance result as statistical significance is questionable. The authors concluded that "An immediate, meaningful reduction in lymphocytic activation may occur after upper cervical high-velocity, low-amplitude thrust."

			Sham (n=3): A deactivated activator instrument was used to deliver sham adjustments to non-dysfunctional joints above or below the determined segments of joint dysfunction.	
Teodorczyk-Injeyan et al 2006	Healthy (n = 64)	TNF-α, IL-1β and substance P	A single session of bilateral hypothenar (Carver-Bridge)–type high-velocity, low-amplitude thrust was applied by a chiropractor to the involved vertebral segment i.e., upper thoracic spine (T1-T6) and was evidenced by an audible cavitation. Participants were initially screened for restrictions in segmental motion in the upper thoracic spine (T1-T6).	Spinal high-velocity, low-amplitude thrust-treated subjects show a time-dependent attenuation of the inflammatory Cytokines (TNF-α, IL-1β) unrelated to systemic levels of substance P.
Teodorczyk-Injeyan et al 2008	Healthy (n = 76)	IL-2 in peripheral blood mononuclear cell cultures	A single session of bilateral hypothenar (Carver-Bridge)–type high-velocity, low-	Anti-inflammatory cytokines IL-2 increased in the intervention group.

			amplitude thrust applied by a chiropractor to the involved vertebral segment i.e., upper thoracic spine (T1-T6), with or without audible cavitation. Participants were initially screened for restrictions in segmental motion in the upper thoracic spine (T1-T6) using motion and static palpation.	
Roy et al 2010	Low back pain (n = 11) and control healthy (n = 10)	IL-6 and CRP	Nine sessions of spinal thrusts were delivered by a chiropractor over two weeks using the Activator IV adjusting instrument. The lumbar area from T12 to L5 was adjusted according to the pelvic-deficient side determined by an Activator Methods Chiropractic Technique evaluation.	IL-6 and CRP decreased after the intervention.
Teodorczyk-Injeyan et al 2010	Healthy (n = 74)	B (CD19), T (CD3) Lymphocytes, immunoglobulin G and	A single high-velocity, low-amplitude thrust, in the form of a bilateral hypothenar push	Spinal high-velocity, low-amplitude thrust did not increase IL-2-dependent polyclonal immunoglobulin synthesis by mitogen-activated B cells. However,

		immunoglobulin M	(Carver Bridge) was delivered by a chiropractor to the involved vertebral segment so as to produce joint cavitation in people who had restricted motion segment in the upper thoracic spine (T1-T6).	antibody synthesis induced by IL-2 alone can be, at least temporarily, augmented following spinal high-velocity, low-amplitude thrust.
Padayachy et al 2010	Acute mechanical low back pain (n=30)	Serum cortisol levels	Group A: A single session of "low back spinal manipulation" was delivered by a chiropractor. No details are given about what the "low back spinal manipulation" encompassed. The exact site of manipulation is not given. Motion palpation was used to identify joint fixations.<br><br>Group B: "subjects were phlebotomized, rested for 5 min and re-phlebotomized. They then	"There was an increase in the rate of change of serum cortisol levels post-low back spinal manipulation although there was a significant decrease in serum cortisol levels between the initial blood sample and the 5-min rest period."

			received a low back spinal manipulation and within 5 min thereafter were re-phlebotomized”	
Puhl & Injeyan 2012	Healthy (randomised n = 56; analysed n=36)	Plasma concentrations of norepinephrine and epinephrine	A single session of combination type thrust (hypothenar transverse push) directed to the hypomobile spinal segment between T1 and T3 or a Carver-Bridge type adjustment (bilateral hypothenar push) for hypomobile segments T4 to T6 was delivered by a chiropractor. The hypomobile spinal motion were identified by using static and motion palpation procedures.	As compared to the control group, there was no significant difference in the mean plasma levels of norepinephrine and epinephrine measured immediately or 15 minutes after an upper thoracic spinal high-velocity, low-amplitude thrust.
Licciardone et al 2012	Non-specific chronic low back pain (n = 55)	Concentrations of IL-1β, IL-6, IL-8, IL-10, and TNF-α	Six sessions of osteopathic manual treatment was given by an osteopath at weeks 0, 1, 2, 4, 6, and 8. Osteopathic manual treatment included high-	As compared to the sham osteopathic manual treatment group, a significant reduction in TNF-α was found in osteopathic manual treatment group when measured four weeks after the last treatment session.

			velocity, low-amplitude thrusts; moderate-velocity, low-amplitude thrusts; soft-tissue stretching; kneading, and pressure; myofascial stretching and release; positional treatment of myofascial tender points; and patient isometric muscle activation against provider unyielding and equal counterforce. It was targeted towards the lumbosacral, iliac and pubic region after standard diagnostic evaluation at each session,	There was no significant effect on concentrations of IL-1β, IL-6, IL-8, and IL-10.
Licciardone et al 2013	Chronic low back pain with diabetes mellitus (n = 6)	TNF-α	Six sessions of osteopathic manual treatment were given by an osteopath at weeks 0, 1, 2, 4, 6 and 8 using an algorithmic approach. Each session lasted 15 minutes. The osteopathic manual treatment protocol consisted of six commonly used	As compared to the sham osteopathic manual treatment, a significant reduction in TNF-α measured four weeks after the last treatment session was observed in osteopathic manual treatment group.

			techniques aimed at the lumbosacral, iliac, and pubic regions. Osteopathic manual treatment techniques were based upon the participant evaluation and their response to the standard protocol. A dirty half dozen framework (lumbar somatic dysfunction, symphysis pubis dysfunction, restriction of the anterior movement of the sacral base, innominate shear dysfunction, short leg and pelvic tilt syndrome, and muscular imbalance of the trunk and lower extremity) was used to assess the participant at each session (Licciardone et al 2008).	
Molina-Ortega et al 2014	Healthy (n = 30)	Substance P and Nitric oxide	Cervical group (n=10): A single session of high-velocity, low-amplitude thrust was applied unilaterally on the	As compared to the control group, there was an increase in substance P plasma level when measured immediately and two hours after cervical high-velocity, low-amplitude thrust (70.55%). No

			posterior joint at the predetermined C5 to C6 vertebral level by a physical therapist. The reason for delivering the thrust to these levels is not given.<br>Thoracic group (n=10): A single session of high-velocity, low-amplitude thrust in anterior to posterior direction was applied with the physical therapist's chest over participants elbow as the participant lied supine with arms crossed over the chest and hands wrapped around the shoulders. The therapist's clenched hand was placed over T4 spinous process. The exact spinal segment and the reason for delivering the thrust to thoracic levels is not given.<br><br>Control group (n=10)	changes in nitric oxide production were observed.

Plaza-Manzano et al 2014	Healthy (n = 30)	Neurotensin, oxytocin, orexin A and cortisol.	Cervical group (n=10): a single session of high-velocity, midrange, left rotational force was delivered by a physical therapist to the mid cervical spine (C4) on the lower cervical spine (C5) in supine, with left rotation and right side bending. The reason for delivering the thrust to these levels is not given.<br>Thoracic group (n=10): a single session of high-velocity, end range, anterior-posterior force was delivered by a physical therapist through the elbows to the middle thoracic spine (T3-4) on the lower thoracic (T4-5) spine in a supine position, with the patient's arms crossed. The reason for delivering the thrusts to these levels is not given.<br>Non manipulation control (n=10)	As compared to the control group, both cervical and thoracic group significantly increased neurotensin and oxytocin levels when measured immediately after the intervention.<br><br>As compared to the control and thoracic group, cervical group significantly increased cortisol plasma levels when measured immediately after the intervention.<br><br>No significant between-group changes in neurotensin, oxytocin, and cortisol were noted when measured two hours after the intervention.<br><br>No significant changes were noted in orexin A levels immediately or after two hours of intervention.

Sampath et al 2017	Healthy men (n = 24)	Salivary cortisol, salivary testosterone, testosterone-cortisol (T/C) ratio, heart rate vari-ability, and changes in oxyhemoglobin concentration of the right calf muscle.	A single high-velocity, low-amplitude thrust was delivered by a physical therapist who placed his hand curled into a fist with the thumb and index finger extended at the level of fifth thoracic vertebra (as this area is known to contain pre-ganglionic neurons of the sympathetic nervous system). The high-velocity, low-amplitude thrust was applied through the participant's upper extremity and thorax upon expiration.	As compared to the sham group, thoracic high-velocity, low-amplitude thrust resulted in an immediate decrease (five minutes postintervention) in salivary cortisol concentration and reduced T/C ratio six hours after intervention.<br><br>Thoracic high-velocity, low-amplitude thrust had no effect on oxyhemoglobin, testosterone or heart rate variability as compared to sham group.
Degenhardt et al 2017	Chronic low back pain (n= 33) and no low back pain control (n= 7)	IL-1β, IL-6, TNF-α, and CRP.	A single session of osteopathic manual treatment was delivered by an osteopath for approximately 20 minutes. The manual treatment comprised of several osteopathic techniques. Indirect and springing techniques were applied on the	There is no significant effect of osteopathic manual treatment on IL-1β, IL-6, TNF-α, and CRP.<br><br>No meaningful difference was noted between low back pain patients and those without low back pain.

			sacrum. The sacroiliac joints were gapped, and kneading was performed on gluteal and lumbosacral erector spinae muscles. Indirect and gentle direct positional release techniques were done to improve lumbar segmental movement. Hypertonic hip flexors and lumbar muscles were stretched by muscle energy techniques. Pubic decompression technique and articulatory sacroiliac joint technique was performed according to the presenting condition of the participants.	
Teodorczyk-Injeyan et al 2018	Nonspecific acute low back pain (n = 19), nonspecific chronic low back pain (n=23) and asymptomatic controls (n=21)	Invitro levels of chemotactic cytokines (chemokine) ligands (CCL2, CCL3, CCL4) and plasma levels of an	Six sessions of high-velocity, low-amplitude thrusts were delivered by chiropractors to the involved segment in the lumbo- sacral region on alternate days over two	After spinal adjustments a significant decline in CCL3 production in both nonspecific acute and chronic low back pain groups. Change scores for CCL4 production differed significantly only for the acute low back pain cohort, and no effect on the production of CCL2 or

		inflammatory biomarker, soluble E-selectin (Se-selectin).	weeks. Thrusts were delivered according to the findings of segmental restriction in the lumbosacral region on a given day and was applied to one segment only (even if the assessment indicated involvement of more than one segment) as indicated by pain or restricted motion on palpation.	plasma E-selectin levels was noted in either group.
Lohman et al 2018	Women with non-specific mechanical neck pain (n=28)	Peripheral blood serum levels of oxytocin, neurotensin, orexin A, and cortisol	A single session of high-velocity, low-amplitude thrust was delivered by the physical therapist with the arc of rotation dependent on the level of target vertebra and performed in both left and right direction, first away from pain then toward pain. The target vertebra was determined based on the site of pain and/or restriction. The exact segment on which	There was significant within-group increase in oxytocin, neurotensin and orexin A for the intervention group. However, there was no significant between- or within-group difference in cortisol levels. There was no significant difference in oxytocin, neurotensin and orexin A levels between the intervention and sham group.

			the thrust was delivered is not given.	
Teodorczyk-Injeyan et al 2021	Acute low back pain (n = 22), chronic low back pain (n = 25), asymptomatic controls (n = 24)	In vitro production of TNF-α, IL-1β, IL-6, IL-2, interferon ɣ (IFNɣ), IL-1 receptor antagonist (IL-1RA), TNF soluble receptor type 2 (sTNFR2) and IL-10	Six sessions of high-velocity, low-amplitude thrusts were delivered by chiropractors in the lumbar or lumbosacral region on alternate days in the span of two weeks. The high velocity, low-amplitude thrust was applied to the involved segment in the form of a spinal push or spinal pull-type adjustment to the lumbar spine, or a sacroiliac adjustment was given according to their findings of segmental restrictions on a given day. Thrusts were delivered to one segment only as indicated by pain or restricted motion palpation.	A general trend towards lowering of the production of pro-inflammatory cytokines (TNF-α, IL-1β, IFNɣ), without statistically significant difference, was noted after the intervention. However, a moderate effect size (Cohen's d > 0.5) for reduced TNF-α production in both patient cohorts and for all other proinflammatory cytokines, except IL-2, in patients with chronic low back pain was found.<br><br>Large intervention-related effect size was noted for enhanced IL-2 production in the acute low back pain group and in reduced IL-6 production in the chronic low back pain group.<br><br>In people with acute low back pain, a significant increase in post-intervention IL-2 levels was observed compared with both the control and people with chronic low back pain.<br><br>In people with chronic low back pain, production of IL-6 significantly reduced after the intervention when compared

				with baseline though it remained slightly elevated compared to controls.<br><br>Pain and disability scores reduced significantly in all low back pain patients, and were positively correlated with IFNɣ and IL-2 levels in the acute low back pain group.
